# *Arid1b* Haploinsufficiency Causes Abnormal Brain Gene Expression and Autism-Related Behaviors in Mice

**DOI:** 10.3390/ijms18091872

**Published:** 2017-08-30

**Authors:** Mihiro Shibutani, Takuro Horii, Hirotaka Shoji, Sumiyo Morita, Mika Kimura, Naomi Terawaki, Tsuyoshi Miyakawa, Izuho Hatada

**Affiliations:** 1 Laboratory of Genome Science, Biosignal Genome Resource Center, Institute for Molecular and Cellular Regulation, Gunma University, 3-39-15 Showa-machi, Maebashi, Gunma 371-8512, Japan; m.shibutani@gunma-u.ac.jp (M.S.); horii@gunma-u.ac.jp (T.H.); msumiyo@gunma-u.ac.jp (S.M.); mikimura@gunma-u.ac.jp (M.K.); terawaki@gunma-u.ac.jp (N.T.); 2 Division of Systems Medical Science, Institute for Comprehensive Medical Science, Fujita Health University, 1-98 Dengakugakubo, Kutsukake-cho, Toyoake, Aichi 470-1192, Japan; hshoji@fujita-hu.ac.jp (H.S.); miyakawa@fujita-hu.ac.jp (T.M.)

**Keywords:** autism spectrum disorder, chromatin remodeling factor, autism spectrum disorder model mouse

## Abstract

Autism spectrum disorder (ASD) is a neurodevelopmental disorder with core symptoms that include poor social communication, restricted interests, and repetitive behaviors. Several ASD mouse models exhibit impaired social interaction, anxiety-like behavior, and elevated perseveration. Large-scale whole exome sequencing studies identified many genes putatively associated with ASD. Like chromodomain helicase DNA binding protein 8 (CHD8), the most frequently mutated gene in individuals with ASD, the candidate gene *AT-rich interaction domain 1B* (*ARID1B*) encodes a chromatin remodeling factor. *Arid1b* heterozygous knockout (hKO) mice exhibited ASD-like traits related to social behavior, anxiety, and perseveration, in addition to associated features reported in some cases of ASD, such as reduced weight, impaired motor coordination, and hydrocephalus. Hydrocephalus was present in 5 of 91 hKO mice, while it was not observed in wild-type littermates (0 of 188). Genome-wide gene expression patterns in *Arid1b* hKO mice were similar to those in ASD patients and *Chd8*-haploinsufficient mice, an ASD model, and to developmental changes in gene expression in fast-spiking cells in the mouse brain. Our results suggest that *Arid1b* haploinsufficiency causes ASD-like phenotypes in mice.

## 1. Introduction

Autism spectrum disorder (ASD) is a neurodevelopmental disorder defined in the Diagnostic and Statistical Manual of Mental Disorders 5 (DSM-5) by three features: impairment of social communication, restricted interests, and repetitive behaviors. Individuals with ASD often have other co-occurring conditions, including epilepsy, depression, anxiety [1,2], attention deficit hyperactivity disorder (ADHD), enlarged lateral ventricle [3], learning disability, and disturbance of motor coordination [4,5,6]. It is estimated that one in 68 children in the United States has an ASD [7].

The results of twin studies suggest that genetic factors play important roles in ASD pathogenesis [8,9]. Although the co-occurrence of ASD in monozygotic twins is higher than that of dizygotic twins, it is not complete, suggesting that environmental factors also contribute [9]. On the basis of these reports, models of autism have been generated using animals genetically modified to mimic the features of ASD patients [10,11,12,13,14,15]. Typical animal models exhibit poor social communication, anxiety, and perseveration [11].

Large-scale whole exome sequencing studies revealed that de novo mutations in many genes contribute to ASD risk. Many of these genes encode proteins involved in synaptic formation, transcriptional regulation, and chromatin remodeling pathways [16,17,18,19,20]. Chromodomain helicase DNA binding protein 8 (CHD8), the most frequently mutated gene in individuals with ASD, encodes a chromatin remodeling factor [21]. *Chd8*-haploinsufficient mice manifest typical autism-like traits, e.g., elevated anxiety, altered social behavior [12,13] and cognitive impairment [14].

*AT-rich interaction domain 1B* (*ARID1B*), another gene mutated in ASD, also encodes a chromatin remodeling factor [22]. In addition to autistic patients, mutations in the *ARID1B* gene are observed in patients with neurodevelopmental delay, intellectual disability, growth delay, and Coffin-Siris syndrome [23,24,25]. No specific region of *Arid1b* is more likely to be mutated in ASD patients [24].

To determine whether this gene is involved in ASD, we used CRISPR/Cas gene editing to generate a deletion in exon 3 of mouse *Arid1b*. Using *Arid1b* heterozygous knockout mice, we investigated whether *Arid1b* haploinsufficiency leads to behavioral, morphological and genetic features related to ASD. This study demonstrates that *Arid1b* haploinsufficiency leads to ASD-like phenotypes in mice.

## 2. Results

### 2.1. Generation of Arid1b Heterozygous Knockout Mice and Assessment of Neurological and Sensory Functions

We generated *Arid1b* knockout (KO) mice by inducing a deletion in exon 3 of the *Arid1b* gene using the CRISPR/Cas system (Figure S1a). This mutation causes a frameshift in translation and premature termination. Mating of male and female heterozygous KO mice (hKO) did not produce any homozygous KO offspring (0 of 19 pups), suggesting that ARID1B is essential for fetal development. *Arid1b* RNA expression in the whole brain in hKO mice was reduced to about 70% of the level in wild-type (WT) littermates (Figure S1b), while the ARID1B protein was reduced to about 50% of that in WT mice (Figure S1c). These data demonstrate that the mutation was successfully introduced in *Arid1b*, and confirm that gene expression was reduced as expected. *Arid1b* hKO male mice in which the mutation originated from the paternal allele were used in subsequent experiments.

Average body weight was significantly lower in hKO mice than in WT mice at 10–13 weeks of age (Figure S2a), which is reminiscent of the smaller body size of ASD patients harboring an *ARID1B* mutation [24]. Body temperature did not differ between hKO and WT mice (Figure S2b). In the hot-plate test, latency of paw responses to a nociceptive stimulus tended to be higher in hKO mice than in WT mice (Figure S2c). In addition, we assessed the response to acoustic stimulus and prepulse inhibition (PPI) of the startle response in the hKO mice (Figure S2d,e). Startle reflex responses caused by 110 dB stimuli were reduced in hKO mice, whereas both hKO and WT mice exhibited startle responses of similar amplitudes to 120 dB stimuli (Figure S2d). In response to a 110 dB stimulus, the hKO mice exhibited levels of PPI stimulus similar to those of their WT littermates, but at 120 dB their PPI was lower than that of the WT mice. These results suggest that hKO mice have mild impairment of the inhibitory process that regulates sensory input to the brain upon presentation of more intense stimuli.

Reduced body weight, sensory defects, and hearing disability have also been reported in ASD patients [24,26]. Thus, *Arid1b* hKO mice exhibited some phenotypes similar to those of ASD patients.

### 2.2. ASD-Like Behaviours in Arid1b hKO Mice

#### 2.2.1. Increased Anxiety-Like Behavior in *Arid1b* hKO Mice

**Figure 1:**
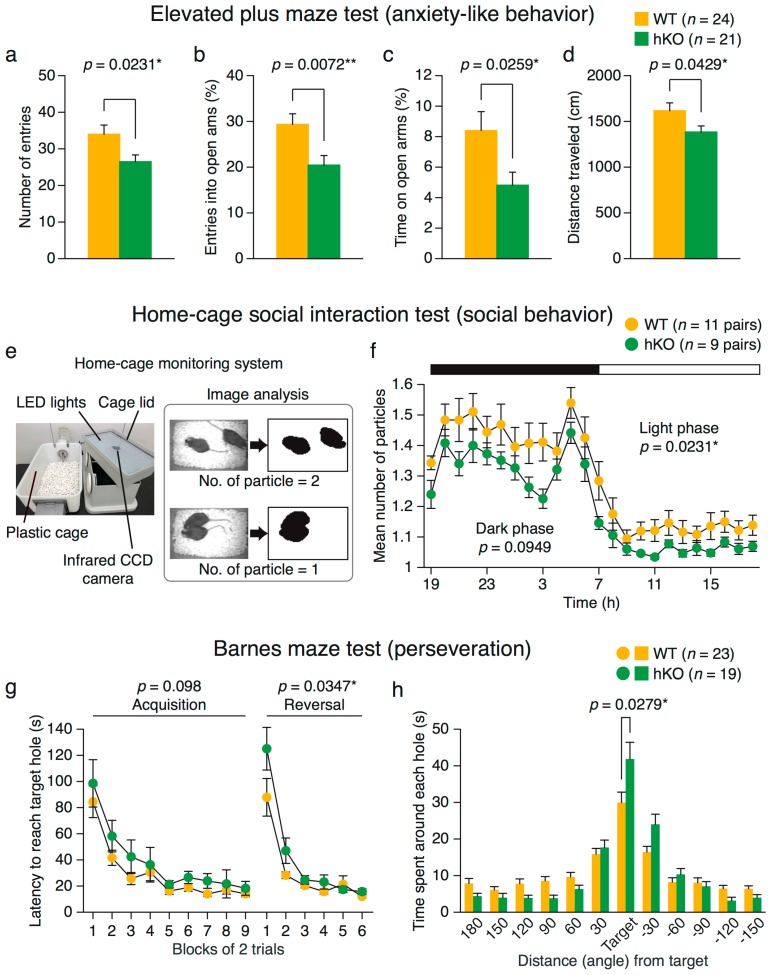
Anxiety-like behavior, social behavior, and reversal learning performance in *Arid1b* hKO mice. (**a**–**d**) Elevated plus maze test; (**a**) number of total entries into arms; (**b**) percentage of entries into open arms; (**c**) stay time in open arms; (**d**) total distance traveled (wild-type (WT) mice, *n* = 24; *Arid1b* hKO mice, *n* = 21); (**e**) home-cage monitoring system for measuring social behavior. The number of particles detected in each image was counted; (**f**) mean number of particles during each hour of the final three days in the apparatus (WT, *n* = 11; *Arid1b* hKO, *n* = 9); (**g**–**h**) Barnes maze test; (**g**) latency of first reaching the target hole across nine blocks of two acquisition trials. One day after the acquisition trials, a probe test was performed in which the target box was removed. After the probe test, the target was located on the opposite side to assess reversal learning; (**h**) stay time around each hole in the probe test held a day after completion of the first training. (WT, *n* = 23; *Arid1b* hKO, *n* = 19). Significant differences between genotypes are indicated by * *p* < 0.05 and ** *p* < 0.01, as determined by Student’s two-tailed *t*-test (**a**–**d**,**h**) or two-way repeated-measures analysis of variance (ANOVA) (**f**,**g**).

We assessed anxiety-like behavior in *Arid1b* hKO mice in the elevated plus maze, light/dark transition, and open field tests (Figure 1a–d and Figure S3). Increased anxiety-like behaviors in hKO mice were observed in the elevated plus maze test (Figure 1a–d). Specifically, *Arid1b* hKO mice showed a lower number of entries into open and closed arms (Figure 1a), a lower percentage of entries into open arms (Figure 1b), and spent less time in open arms (Figure 1c). Total distance traveled was also significantly lower in the hKO group (Figure 1d). In the light/dark transition test, no significant differences were observed in terms of total distance traveled (Figure S3a), stay time in the light box (Figure S3b), number of transitions (Figure S3c), or latency to the light box (Figure S3d). In the open field test, *Arid1b* hKO mice exhibited reductions in exploratory behaviors, including distance traveled (Figure S3e) and vertical activity (Figure S3f), and stereotypic counts that possibly reflect repetitive behaviors (Figure S3h), whereas time spent in the center area, an index of anxiety, did not differ between hKO and WT mice (Figure S3g). Overall, these results suggest that certain types of anxiety-like behavior are increased in hKO mice.

#### 2.2.2. Altered Social Behavior in *Arid1b* hKO Mice

We evaluated social behavior in the home cage using a home-cage monitoring system (Figure 1e). Two mice of the same genotype, previously housed separately, were placed into the cage for a week and monitored with a video camera. The number of particles in each image provides a measurement of contact: when the mice were not in contact with each other, they were detected separately (two particles); whereas when the mice were in contact with each other, they were detected as a single object (one particle) (Figure 1f and Figure S4a). The average number of particles during each hour of the last 3 days was reduced in the hKO group (Figure 1f and Figure S4a), whereas overall activity levels did not differ between groups (Figure S4c). These results indicate that *Arid1b* hKO mice exhibited altered social behavior in the home cage. By contrast, in a novel environment, their behaviors were similar to that of WT, as shown by the social interaction test (Figure S5a–e) and three-chamber test (Figure S5f–i).

#### 2.2.3. Perseveration (Behavioral Inflexibility) in *Arid1b* hKO Mice

Perseveration (impaired cognitive and behavioral flexibility) is a characteristic of autism [24]. To evaluate behavioral flexibility, mice were first trained to find and enter the target box in the Barnes maze during an acquisition session, and then subjected to the reversal task (Figure 1g,h). Arid1b hKO and WT mice exhibited similar latency in reaching the target across acquisition sessions (Figure 1g, left), and in the probe trial 24 h after the acquisition, hKO mice spent more time around the target hole than WT mice (Figure 1h). These results suggest not only normal spatial learning ability but also improved memory performance in hKO mice. Moreover, hKO mice exhibited elevated perseveration, as indicated by their longer latency in reaching a target box placed in the opposite location during the reversal task (Figure 1g, right).

#### 2.2.4. Impaired Motor Coordination in *Arid1b* hKO Mice

**Figure 2:**
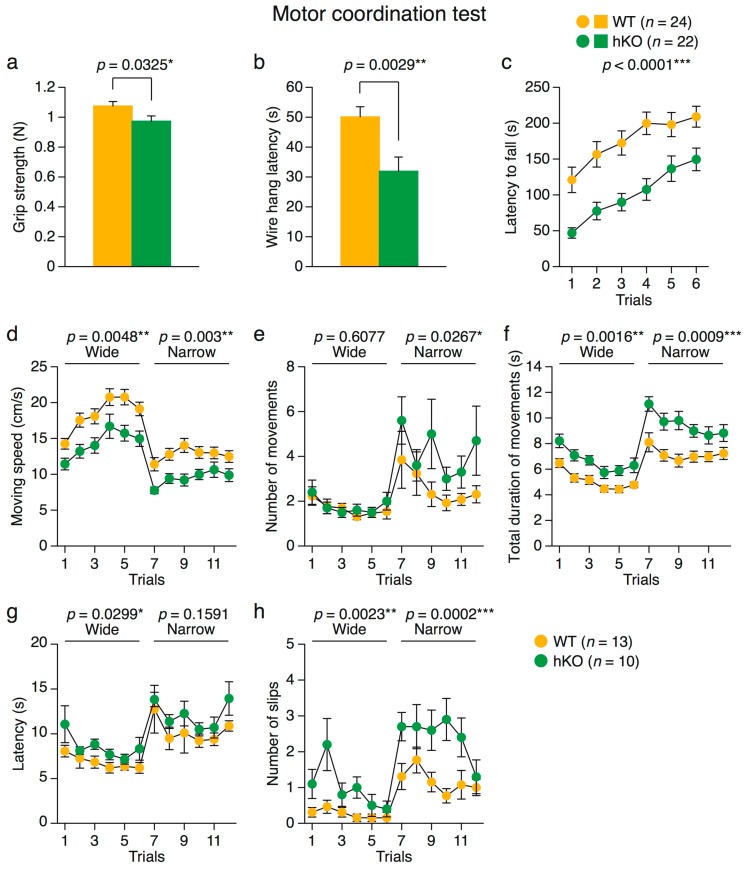
Muscular strength and motor coordination in *Arid1b* hKO mice. (**a**) Forelimb grip strength; (**b**) latency of falling from the wire mesh in the wire hang test; (**c**) latency of falling from the rotarod (WT, *n* = 24; *Arid1b* hKO, *n* = 22); (**d**–**h**) beam test; (**d**) movement speed; (**e**) number of movements; (**f**) total duration of movements; (**g**) latency of crossing the beam; (**h**) number of slips. (WT, *n* = 13; *Arid1b* hKO, *n* = 10). Mice falling off the beam in the beam test were excluded from the analysis. Significant differences between genotypes are indicated by * *p* < 0.05 and ** *p* < 0.01, *** *p* < 0.001 as determined by Student’s two-tailed *t*-test (**a**,**b**) or two-way repeated-measures ANOVA (**c**–**h**).

Another feature observed in some ASD patients is a deficit in motor coordination [4,6]. Hence, we assessed muscular strength and motor coordination in *Arid1b* hKO mice (Figure 2). Grip strength and the latency of falling off the wire mesh in the wire hang test were significantly lower in the hKO mice (Figure 2a,b). In the rotarod test, the latency of falling off the accelerating rotating rod was significantly lower in hKO mice than in WT mice (Figure 2c). In the balance beam test, *Arid1b* hKO mice exhibited slower movement speed (Figure 2d), longer duration of movement (Figure 2f), and a higher number of slips when crossing wide or narrow beams (Figure 2h). The number of movements was significantly higher on the narrow beam (Figure 2e), and the latency of reaching the goal was significantly larger on the wide beam (Figure 2g) in hKO mice. Together, these results indicate that the hKO mice had reduced muscular strength and deficits in motor coordination.

### 2.3. Other Behavioural Features in Arid1b hKO Mice

#### 2.3.1. Heightened Response to Intense Aversive Stimuli in *Arid1b* hKO Mice

**Figure 3:**
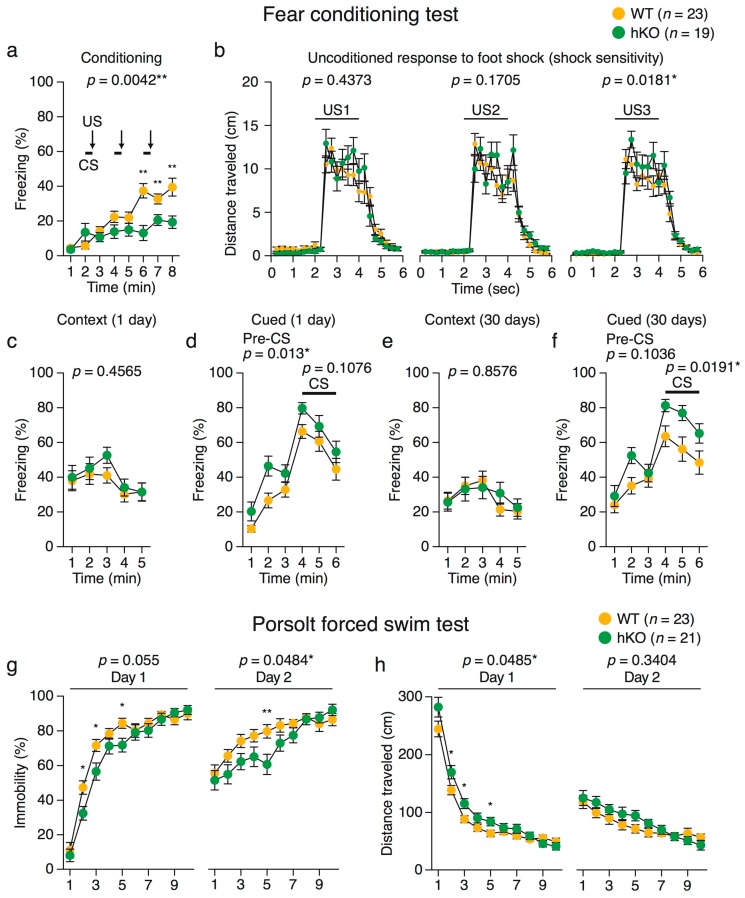
Fear memory and depression-like behavior in *Arid1b* hKO mice. (**a**–**f**) Fear conditioning test; (**a**) percentage of freezing in the conditioning session, in which both conditioned stimulus (CS; white noise) and unconditioned stimulus (US; foot shock) were presented; (**b**) distance traveled during and after foot shocks; (**c**,**d**) context and cued tests one day after conditioning; (**e**,**f**) context and cued tests 30 days after conditioning; (**g**,**h**) Porsolt forced swim test; (**g**) percentage of immobility time on Days 1 and 2; (**h**) Distance traveled on Days 1 and 2. Data in the fear conditioning test (WT, *n* = 23; *Arid1b* hKO, *n* = 19) and the Porsolt forced swim test (WT, *n* = 23; *Arid1b* hKO, *n* = 21) are expressed as the mean ± SEM. Significant differences between genotypes are indicated with * *p* < 0.05 and ** *p* < 0.01, as determined by two-way repeated-measures ANOVA (**a**–**h**).

Although the hKO mice exhibited impaired motor performance and reduced locomotor activity in novel environments (Figure 1a–d, Figure 2 and Figure S3e,f,h), exposure to intense stimuli such as electric foot shock or forced swim stress induced larger locomotor responses in hKO mice than in WT mice (Figure 3). In the fear conditioning test, hKO mice traveled longer distances both during and immediately after foot shock, an index of pain sensitivity (Figure 3b). Moreover, hKO mice were more active than WT mice in the Porsolt forced swim test (Figure 3h). These results indicate that hKO mice had an increased response to strong stimuli such as electro-shock and water immersion.

#### 2.3.2. Enhanced Fear Generalization and Long-Term Fear Memory in *Arid1b* hKO Mice

Fear memory was assessed in the contextual and cued fear conditioning test. *Arid1b* hKO mice exhibited less freezing during the conditioning than WT mice (Figure 3a,b), whereas hKO and WT mice exhibited similar levels of freezing in the context test (Figure 3c) and hKO mice exhibited more freezing in the altered context in the absence of a conditioned stimulus (CS) (Figure 3d). Increased levels of freezing were observed 30 days after the conditioning session (Figure 3e,f). These results suggest an increase in fear generalization and long-term fear memory in *Arid1b* hKO mice. In the cued test, the freezing time of hKO mice was increased without CS on the day after conditioning (Figure 3d), and the tendency was maintained for 30 days (Figure 3f, before CS), whereas the ratios in the context test did not differ between hKO and WT mice (Figure 3c,e). Freezing time caused by CS in the cued test (30 days after conditioning) was longer in hKO mice than in WT mice (Figure 3f, CS), suggesting that fear memory was fixed more strongly in hKO mice.

### 2.4. Genome-Wide RNA Expression Profile in Arid1b hKO Mice

**Figure 4:**
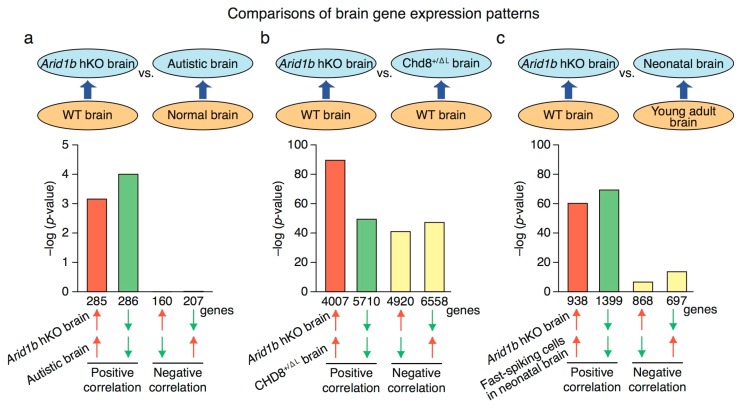
Comparison of brain gene expression patterns in *Arid1b* hKO mice with other genomic data. (**a**) Comparison of brain gene expression patterns in *Arid1b* hKO mice with those in autistic patients; (**b**) *Chd8*-haploinsufficient mice, and (**c**) fast-spiking cells in 7-day-old mouse pups. Arrows show up regulated gene group (red) or down regulated gene group (green).

Next, we performed RNA-Seq and gene expression pattern analyses to obtain genome-wide RNA expression profiles of the brains of hKO and WT mice. We then compared the expression patterns of hKO mice and ASD patients using BaseSpace (Illumina) (Figure 4). This analysis revealed that the gene expression pattern in the hKO mouse brain was similar to that in the brains of autistic patients (Figure 4a), but not to that in the brains of patients with other disorders (Figure S6). As noted above, the chromatin remodeling factor CHD8 is strongly associated with ASD [12], and ARID1B is also involved in chromatin remodeling. Hence, we compared brain gene expression profiles between *Arid1b* hKO and *Chd8* hKO mice and found that they were similar (Figure 4b). Individuals with ASD, and ASD mouse models, exhibit delayed neurodevelopment and disruption of inhibitory brain circuits [27]. Fast-spiking cells (parvalbumin-expressing inhibitory neurons) play essential roles in brain development [27]. Hence, to assess neurodevelopment of the inhibitory brain circuit in *Arid1b* hKO mice, we compared gene expression in hKO mice with developmental gene expression changes in fast-spiking cells. Indeed, the gene expression pattern of *Arid1b* hKO mouse brain was similar to the pattern observed in immature fast-spiking cells (Figure 4c).

### 2.5. Hydrocephalus in Arid1b hKO Mice

Individuals with hydrocephalus develop behavioral problems and autistic symptoms [3]. Some hKO mice (5 of 91) exhibited severe hydrocephalus (Figure S7), whereas none of the littermate WT mice did (0 of 188). The result suggests that hydrocephalus occurs significantly in *Arid1b* hKO mice (*p* = 0.0041, Fisher’s exact test). hKO mice with inflated heads (arrowed in Figure S7a) had reduced body weight, not only relative to WT littermates (11.4 ± 2.7 g vs. 22.2 ± 1.7 g, mean ± S.D., in hKO mice with inflated head vs. WT, respectively; 8–9 weeks of age; *p* < 0.01, Student’s two tailed *t*-test), but also relative to hKO littermates without inflated heads (19.3 ± 2.5 g, *p* < 0.05). Sagittal sections of brains from mice with hydrocephalus are shown in Figure S7b,c. In comparison with the brains of WT mice (Figure S7b), the brains of these hKO mice had an enlarged lateral ventricle (Figure S7c, arrow), a cavity with new vascularization (Figure S7d, shown in the left red frame in Figure S7c), and corruption of the pyramidal cell layer of the hippocampus (Figure S7e, shown in the right red frame in Figure S7c). No histological abnormalities were observed in hKO mice without hydrocephalus, suggesting that the abnormal behavior of hKO mice revealed by the behavioral analyses could not be assigned to detectable histological abnormalities in their brains.

## 3. Discussion

Typical features of ASD patients include impaired social communication, anxiety, repetitive behavior, and restricted interests [1,2,24]. ASD-like model mice exhibit similar traits, including anxiety-like behavior, impaired social behavior, and perseveration (behavioral inflexibility) [10,11,12]. The presence of these behavioral phenotypes is an important criterion for evaluating the relevance of a mouse model to autism [15]. Here, we observed these core features in *Arid1b* hKO mice, which therefore represent a novel ASD-like model.

*Arid1b* hKO mice manifested anxiety-like behavior in the elevated plus maze test, and reduced exploration in the open field test. *Arid1b* hKO mice exhibited increased contact with each other in the home-cage social interaction test. Previous work shows that interpersonal distance is reduced in ASD patients [28]. Consistent with this, *Chd8* mutant mice, an established ASD model, also exhibit elevated contact time [12]. Both CHD8 and ARID1B are involved in chromatin remodeling. We also evaluated social behavior in the social interaction and three-chambers tests, but observed no significant differences between *Arid1b* hKO and WT mice. These results suggest that the impairment of social behavior in *Arid1b* hKO mice is mild. It is notable that increased social interaction was observed only in the home-cage social interaction test. The differences in the results between the home-cage social interaction test and the other tests for assessing social behavior may have been caused by the difference in measurement time; the home-cage social interaction test was a one-week test session whereas the social interaction test in a novel environment and the three-chamber social approach test were 10-min test sessions. Social behavior in *Arid1b* hKO mice might be counteracted by their increased anxiety when exposed to a novel environment or unfamiliar conspecific mouse, resulting in similar levels of social behavior to that in WT mice until they are habituated to the testing situation in social behavioral tests. Inflexibility in switching tasks (perseveration) is often observed in ASD patients [24], and *Arid1b* hKO mice exhibited perseveration in the Barnes maze test. By contrast, although intellectual disabilities have been reported in ASD patients [29], the *Arid1b* hKO exhibited no impairment in learning ability or memory in the Barnes maze, fear conditioning, and T-maze tests (data not shown). Many autism risk genes overlap with the genes responsible for intellectual disability [30,31], but these results indicate that *Arid1b* mutation is not associated with this type of impairment.

Celen et al. recently reported that *Arid1b* haploinsufficient mice exhibit a short stature and hydrocephalus [32] as observed in our mice, although there were some differences in behavioral features between their mice and our mice. Their results showed that *Arid1b* haploinsufficient mice engage in increased anxiety-like behavior and reduced social behavior, whereas the results of our study revealed that *Arid1b* haploinsufficient mice show behavioral changes reminiscent of those of ASD patients, although the behavioral abnormalities seem to be mild (Table 1). The differences in behavioral phenotypes between the two lines of *Arid1b* hKO mice may reflect the behavioral diversity of ASD. Alternatively, the differences could be because the deleted region of *Arid1b* was different between the two lines; the mutation was induced in exon 3 in our mice, whereas exon 5 was deleted in their mice.

Associated symptoms in some ASD patients include sensory disorders, difficulties in motor coordination [4,6], hydrocephalus [3], and reduced body weight [24]. The body weight of *Arid1b* hKO mice was significantly reduced at 10–13 weeks of age, which is reminiscent of the short stature and reduced body weight of ASD patients with a mutation in *ARID1B* [24]. Patients with Coffin-Siris syndrome, which is associated with the *ARID1B* mutation, also have short stature [25]. These reports and our data suggest that the *ARID1B* mutation causes short stature and Coffin-Siris syndrome. In addition, *Arid1b* hKO mice showed reduced muscular strength, impaired motor coordination, a tendency to reduced pain sensitivity, and poorer hearing; 5% of *Arid1b* hKO male mice had enlarged brain ventricles. Together, these data show that the physiological and behavioral features of *Arid1b* hKO mice are similar to the core and associated symptoms observed in ASD patients.

We observed a heightened response to strong stimuli such as electro-shock and water invasion. This feature has not been reported previously in autism model mice, but could be related to the hypersensitivity to sensory stimuli observed in ASD patients [26]. The hKO mice also exhibited a heightened fear memory, which has been observed in previous animal models of autism [33].

We compared the brain gene expression patterns of *Arid1b* hKO mice with those of ASD patients and *Chd8* hKO mice, and identified extensive overlap in both comparisons. Because both *Arid1b* and *Chd8* encode chromatin remodeling factors, the phenotypes common to *Arid1b* and *Chd8* hKO mice, such as the alterations in social interaction, might be the consequence of common changes in brain expression profiles, and the associated mechanisms could underlie the symptoms of ASD. We also found that gene expression in the *Arid1b* hKO mouse brain resembled the pattern in immature fast-spiking cells, which is observed in autistic brains [27].

Together, our results show that *Arid1b* haploinsufficiency in mice leads to abnormal brain gene expression and behaviors related to ASD. Therefore, *Arid1b* hKO mice represent a useful tool for advancing our understanding of the pathogenesis and underlying mechanisms of neurodevelopmental disorders.

## 4. Materials and Methods

### 4.1. Animals

All animal procedures were approved by the Animal Care and Experimentation Committee at Gunma University (14-048, 27 August 2014) and Fujita Health University (I0741, 30 November 2015), and carried out in accordance with approved guidelines.

*Arid1b* KO mice were generated as previously described [34], except that the double-nicking method was used during genome editing [35]. In brief, female C57BL/6J mice (CLEA Japan, Inc., Tokyo, Japan) were super-ovulated by intraperitoneal injection of 7.5 units of pregnant mare’s serum gonadotropin (PMSG; ASKA Pharmaceutical Co., Ltd., Tokyo, Japan), followed by 7.5 units of human chorionic gonadotropin (hCG; ASKA Pharmaceutical) 48 h later. The females were mated overnight with C57BL/6JJcl male mice, and fertilized embryos were collected from the oviducts the next day. Cas9D10A and gRNAs targeting exon 3 (5′-CCAGGCACAGTACCTGCAGC-3′ and 5′-GGCGGGAGGTGCGAGGGCTG-3′) were injected into the cytoplasm of fertilized embryos, which were then cultured in KSOM with amino acids at 37 °C in 5% CO_2_/95% air.

To generate mutant mice, one-cell embryos were transferred into the oviduct ampulla (9–18 embryos per oviduct) of pseudopregnant jcl:ICR (CLEA Japan, Inc., Tokyo, Japan) females. Genotyping and sequencing were performed using primers 5′-TGCTCTATGTAACTTTCTGACAGATG-3′ and 5′-CTGCTGTGGCTGGTACCTCT-3′.

Male *Arid1b* hKO or WT mice used in the behavioral tests were generated by mating *Arid1b* hKO male mice with C57BL/6J female mice. The parental hKO mice were backcrossed into C57BL/6J for over three generations. Mice were subjected to a battery of behavioral tests (Table S1) from 10 to 42 weeks of age. The behavioral tests were performed between 09:00 and 18:00.

### 4.2. General Procedures for Behavioral Tests

Male hKO and WT mice were housed in groups of four per cage (two KO and two WT animals per cage). Mice with hydrocephalus were not used for the behavioral tests. The mice were kept in a room with a 12 h light/dark cycle (lights on between 07:00 and 19:00) and allowed free access to food and water. Behavioral tests were performed between 09:00 and 18:00 when the mice were 10–42 weeks of age. Test sequence of the behavioral test battery is shown in Table S1.

### 4.3. Motor Function Tests

A wire hang test was carried out to assess neuromuscular strength. The apparatus consisted of a box (21.5 cm × 22 cm × 23 cm) with a wire-mesh grid ceiling (10 cm × 10 cm) that can be inverted. At 10–13 weeks of age, mice were placed on the wire mesh, which was then inverted, forcing the animals to hang on the wire. The latency of falling from the wire was recorded with a 60 s cutoff time.

Motor coordination and balance were tested using the rotarod and beam tests. For the former, mice (12–15 weeks of age) were placed on a rotating drum with a diameter of 3 cm (Accelerating Rotarod; UGO Basile, Comerio, Italy), and the time that each mouse was able to maintain its balance on the rod during acceleration from 4 to 40 rpm over 5 min was recorded.

The beam test was carried out using two 1 m rods of 28 mm (wide) or 11 mm (narrow) in diameter. Motor coordination was assessed by movement speed and the number of pauses and slips as each mouse walked along each rod. After six trials on the wide rod, six trials were carried out on the narrow rod. The mice used for this experiment were 16–19 weeks of age.

### 4.4. Hot Plate Test

The hot-plate test was used to evaluate sensitivity to a painful stimulus. Mice (12–14 weeks of age) were placed on a hot plate at 55.0 ± 0.3 °C (Columbus Instruments, Columbus, OH, USA), and the latency of the first paw response (foot shake or paw lick) was recorded with a cutoff time of 15 s.

### 4.5. Open Field Test

Mice (11–13 weeks of age) were placed in the corner of an open-field apparatus (40 cm × 40 cm × 30 cm; Accuscan Instruments Inc., Columbus, OH, USA), which was illuminated at 100 lx. Total distance traveled (cm), vertical activity (measured by counting the number of infrared beam interruptions), time spent in the central area (20 cm × 20 cm), and number of stereotyped behaviors (defined by the number of breaks of the same infrared beam) were recorded over 120 min.

### 4.6. Light/Dark Transition Test

The apparatus for the light/dark transition test consisted of a cage (21 cm × 42 cm × 25 cm) divided into light and dark chambers, separated by a doorway (O’Hara & Co., Tokyo, Japan). Mice (10–13 weeks of age) were placed on the dark side and allowed to move freely between the two chambers. Latency of first entry into the light chamber (s), distance travelled (cm), and time spent in each chamber (s) were recorded using the ImageLD software (available online: http://www.mouse-phenotype.org/software.html).

### 4.7. Elevated Plus Maze Test

The apparatus consisted of two open arms (25 cm × 5 cm) and two enclosed arms of the same size equipped with transparent walls 15 cm high (O’Hara & Co.). The arms and central square (5 cm × 5 cm) were made of white plastic and elevated to a height of 55 cm above the floor. To prevent mice from falling off the arms, the open arms were surrounded by a raised ledge (2 mm thick, 3 mm high). Arms of the same type were arranged on opposite sides. Mice (11–14 weeks of age) were placed in the central square of the maze facing one of the closed arms and behavior recorded for 10 min. The number of entries into each arm, time spent in each arm (s), and distance traveled (cm) were measured using the ImageEP software (available online: http://www.mouse-phenotype.org/software.html).

### 4.8. Social Interaction Test in a Novel Environment

In the social-interaction test, two mice (12–14 weeks of age) of the same genotype that had been housed in different cages were placed together in a box (40 cm × 40 cm × 30 cm) and allowed to explore freely for 10 min. The total number of contacts, total duration of contacts, mean duration per contact, total duration of active contacts, and distance traveled (cm) were measured using Image SI software(available online: http://www.mouse-phenotype.org/software.html). Images were captured at a rate of three frames per second using a charge-coupled device (CCD, WAT-902B, Watec Co., Ltd., Yamagata, Japan) camera attached above the box, and the distance traveled between successive frames was determined for each mouse. When the two mice contacted each other and previous distance between them was over 10 cm, the behavior was considered as an active contact.

### 4.9. Three-Chamber Social Approach Test

The three-chamber social approach test was performed to investigate sociability and preference for social novelty in mice, as previously described [12]. The apparatus consisted of a rectangular, three-chambered box and a lid with a video camera (O’Hara & Co., Tokyo, Japan). Each chamber (20 cm × 40 cm × 47 cm) was divided by a clear plastic wall with a small square opening (5 cm × 3 cm). First, each subject mouse was placed in the box and allowed to explore for 10 min to habituate the environment. During the session, an empty wire cage (9 cm in diameter, 11 cm in height, with vertical bars 0.5 cm apart) was located in the corner of each chamber. Next, an unfamiliar C57BL/6J male mouse (stranger 1) that had had no prior contact with the subject mouse was put into a wire cage that was placed into one of the side chambers. To assess sociability, the subject mouse was allowed to explore the box for an additional 10-min session. Finally, to evaluate social preference for a new stranger, a second stranger male mouse (stranger 2) was placed into the wire cage that had been empty during the first 10-min session (social novelty preference test). Thus, the subject mouse had a choice between the first, now-familiar mouse (stranger 1) and the novel unfamiliar mouse (stranger 2). The time spent in each chamber and the time spent around each cage was calculated from video images using the ImageCSI software (available online: http://www.mouse-phenotype.org/software.html).

### 4.10. Home-Cage Social Interaction Test

The home-cage monitoring system consisted of a home cage and a cage top with an infrared video camera (25 cm × 15 cm × 23.5 cm, interior dimensions). Two mice of the same genotype that had been housed separately were placed together in the home cage. Images from each cage were captured at a rate of 1 frame per second. Social interaction was measured by counting the number of particles detected in each frame (two particles indicated that the mice were not in contact with each other, whereas one particle indicated contact). The activity level of the mice was also measured by quantifying the number of pixels that changed between each pair of successive frames. The measurements were automatically performed for 1 week using the ImageHA software (available online: http://www.mouse-phenotype.org/software.html).

### 4.11. Barnes Maze Test

The test was conducted on a white circular platform (100 cm in diameter) with 12 holes spaced equally around the perimeter (O’Hara & Co., Tokyo, Japan). The circular maze was elevated 75 cm from the floor. A black escape box (17 cm × 13 cm × 7 cm) with cage bedding paper was located under one of the holes. The hole above the escape box represented the target hole. The location of the target was consistent for a given mouse but was randomized across mice. To prevent bias based on olfactory or proximal cues within the maze, the maze was rotated daily, with the spatial location of the target unchanged with respect to distal visual room cues. Mice were given one to two trials per day for 11 successive days (18 trials in total) to acquire the target location (acquisition session). On Day 12, a probe trial was conducted without the escape box to confirm that this spatial task was acquired based on navigation using distal environment room cues. After the probe trial, mice were given two additional acquisition trials to prevent extinction. The following day after the last trial, mice were subjected to the reversal learning task in which the escape box was relocated 180° from its original position (reversal session) to assess. The latency of reaching the target hole and the time spent around each hole were recorded using the ImageBM software available online: http://www.mouse-phenotype.org/software.html).

### 4.12. Startle Response and Prepulse Inhibition Tests

A startle-reflex measurement system (O’Hara & Co., Tokyo, Japan) was used to measure startle response and prepulse inhibition. Mice (13–16 weeks of age) were placed in a Plexiglas cylinder within a sound-attenuating chamber and left undisturbed for 10 min. White noise (40 ms) was used as the startle stimulus. The startle response was recorded for 400 ms, starting with the onset of the startle stimulus. The peak startle amplitude was used for analysis. The background noise level in the chamber was 70 dB. Each test session consisted of six trial types, two types of startle stimulus-only trials and four types of prepulse inhibition trials. The intensity of the startle stimulus was 110 or 120 dB. The prepulse sound (74 or 78 dB) was presented 100 ms before the startle stimuli. Four combinations of prepulse and startle stimuli were applied (74 dB prepulse + 110 dB pulse, 78 dB prepulse + 110 dB pulse, 74 dB prepulse + 120 dB pulse, and 78 dB prepulse + 120 dB pulse). Six blocks of six trial types were presented in pseudorandom order such that each trial type was presented once within a block. The average inter-trial interval was 15 s (range, 10–20 s).

### 4.13. Porsolt Forced Swim Test

Mice (13–16 weeks of age) were placed in a clear cylinder (20 cm height × 10 cm diameter, O’Hara & Co., Tokyo, Japan) filled with water (approximately 23 °C) to a height of 7.5 cm. The immobility time (as a percentage of total time) and distance traveled (cm) were recorded for 10 min using the ImagePS software (available online: http://www.mouse-phenotype.org/software.html).

### 4.14. Fear Conditioning Test

Mice (33–36 weeks of age) were placed in a conditioning chamber (26 cm × 34 cm × 29 cm) and allowed to explore freely for 2 min. After a 2-min exploration period, an auditory cue (55 dB white noise) was presented for 30 s as a conditioned stimulus (CS). During the last 2 s of the CS, the mice were given a mild foot shock (0.3 mA, 2 s) as an unconditioned stimulus (US). Two more CS–US pairings were presented at 120 s intervals. Approximately 24 h after the conditioning, a context test was performed in the conditioning chamber. In addition, a few hours after the context test, a cued test in an altered context was performed in a triangular box (35 cm × 35 cm × 40 cm) made of white opaque plastic, which was located in a different room. In the cued test, after the initial 3-min period of no CS presentation, the CS was presented during the last 3-min period of the test. The context and cued tests were also performed 30 days after conditioning to evaluate long-term memory. The percentage of freezing time and distance traveled (cm) were calculated using the ImageFZ software (available online: http://www.mouse-phenotype.org/software.html), as previously described [36].

### 4.15. RNA-Seq Analysis

Total RNA was isolated from whole brains of male hKO and WT mice (*n* = 4 of each genotype) using Trizol (Life Technologies, Carlsbad, CA, USA). Stranded cDNA libraries from each total RNA sample were prepared using NEBNext Ultra Directional RNA Library Prep Kit for Illumina (New England Biolabs, Ipswich, MA, USA). The quality of the libraries was assessed using an Agilent 2200 TapeStation, High Sensitivity D1000 (Agilent Technologies, Santa Clara, CA, USA). Pooled libraries generated from the samples were sequenced on an Illumina HiSeq system as 125-base-pair (bp) paired-end reads. Sequencing adaptors, low-quality reads, and bases were trimmed using the Trimmomatic-0.32 tool [37]. The sequence reads were aligned to the mouse reference genome (MMG) using TopHat 2.0.13 (bowtie2-2.2.3; Available online: http://bowtie-bio.sourceforge.net/bowtie2/index.shtml) [38], which can align reads onto their locations (including splice sites) in the genome sequence. Files of the gene model annotations and known transcripts, which are necessary for the whole transcriptome alignment with TopHat, were downloaded from Illumina’s iGenomes website (Available online: http://support.illumina.com/sequencing/sequencing_software/igenome.html). The aligned reads were subjected to downstream analyses using the StrandNGS 2.6 software (Agilent Technologies). Read counts allocated for each gene and transcript (UCSC version 2012.12.16; Available online: http://genome.ucsc.edu/cgi-bin/hgGateway?redirect=manual&source=genome.ucsc.edu) were quantified using the Trimmed Mean of M-value (TMM) method [39].

### 4.16. Gene Expression Pattern Analysis

Genome-wide gene expression profiles were compared using the BaseSpace Correlation Engine (BSCE; formally known as NextBio; available online: https://japan.ussc.informatics.illumina.com/c/nextbio.nb; Illumina, Cupertino, CA, USA) as previously described [40,41]. In brief, the RNA-Seq dataset from hKO mouse brains was compared with three publicly available microarray datasets: autistic patients (GSE38322) [42], *Chd8* mutant mice (DRA003116) [12], and neonatal brain (GSE17806) [43]. Genes with *p*-values < 0.05 and absolute fold changes >1.2 were classified as differentially expressed genes in each dataset.

The processed datasets were compared using BSCE, followed by the Running Fisher test [44] to evaluate the pairwise correlations (overlap) between any two datasets (Table S2).

### 4.17. Quantitive Reverse Transcription Polymerase Chain-Reaction (RT-PCR) Analysis

Total RNA was isolated as described above for RNA-Seq analysis. Gene expression levels were measured with a LightCycler 96 (Roche Holding AG, Basel, Switzerland) using SYBR Premix Ex Taq (Takara Bio Inc., Kusatsu, Japan) according to the manufacturer’s instructions. Primer sequences were 5′-TGGCTCCGTAGAAGCATCA-3′ and 5′-ACCCGATTTAAGGGACATCA-3′ for *Arid1b* and 5′-AATGCATCCTGCACCACCAA-3′ and 5′-GTGGCAGTGATGGCATGGAC-3′ for *Gapdh*. Expression levels were normalized to the level of *Gapdh*.

### 4.18. Western Blot Assay

Whole brains were collected from male hKO and WT mice (*n* = 4 of each genotype) and mushed and lysed in radioimmunoprecipitation assay (RIPA) buffer containing protease inhibitors. Protein concentration in each sample lysate was adjusted to 0.5 μg/μL with 2× sample buffer. An equal amount volume of each lysate (10 μL) containing 5 µg protein was loaded onto Extra PAGE One precast gels (7.5%, Nacalai Tesque, Kyoto, Japan). Proteins were transferred to a polyvinylidene difluoride (PVDF) membrane using WSE-7210 EzFastBlot HMW (Atto). The membrane was blocked with 5% ECL Prime blocking agent (GE) and incubated with primary antibody, anti-ARID1B (ab57461, abcam) or anti-TUBULIN (PM054, MBL), followed by secondary antibody, either anti-Mouse IgG HRP (#7076, CST) or anti-Rabbit IgG HRP (NA934VS, GE), respectively. The bands were visualized with ECL Prime (GE). Band intensities were quantified by densitometry using the ImageJ software and the quantity of ARID1B protein in each sample was normalized to that of TUBULIN.

### 4.19. Histology of Arid1b hKO Mouse Brain

Brains of *Arid1b* hKO mice with hydrocephalus and their littermates (at 8–12 weeks of age) were prepared for morphological analysis. Mice were euthanized by cervical dislocation and decapitated. Brains were removed quickly and fixed overnight with 4% paraformaldehyde in 0.1 M phosphate buffer (pH 7.4). The fixed brains were embedded in paraffin, sagittally sectioned (5-µm thick) on a microtome, and mounted on slides. The brain sections were stained with hematoxylin and eosin and morphologically examined under a microscope.

### 4.20. Statistical Analyses

Behavioral data were obtained using applications based on the public-domain ImageJ program and modified for each test by Tsuyoshi Miyakawa (ImageLD, ImageEP, and ImageFZ are freely available online: http://www.mouse-phenotype.org/software.html). Statistical analyses were performed using SAS University Edition (SAS Institute). The data were analyzed using the two-tailed unpaired Student’s *t*-test, paired Student’s *t*-test, one-way analysis of variance (ANOVA), or two-way repeated-measures ANOVA followed by a post-hoc least significant difference test, as appropriate. The significance level was set at *p* < 0.05. Values in graphs are expressed as the mean ± SEM. Details were summarized in Table S3.

### 4.21. Data Availability

Sequencing data have been deposited in the DNA Data Bank of Japan sequence read archive and now under process of accession number assignment. Down regulated and up regulated genes were indicated in supplemental Tables (Tables S4 and S5).

## Figures and Tables

**Table 1:** Comparison of our mice with those of previously reported *Arid1b* haploinsufficient mice (Celen et al., 2017 [32]).

Features	Behavioral Test	Our Study	Celen et al. [32]
Intellectual/cognitive disability	Morris water maze test	Unknown	No
	Barnes maze test	No	Unknown
	T-maze spontaneous alternation test	No	Unknown
	Fear conditioning test	Altered	No
Growth retardation		Yes	Yes
Coarse facial features		Unknown	Unknown
Brachydactyly, hypoplastic nail/finger		Unknown	Unknown
Muscle hypotonia		Yes	Yes
Hydrocephalus		Yes (5.5%)	Yes (6.6%)
Abnormal vocalization		Unknown	Yes
Anxiety	Open field test	Not clear	Yes
	Elevated plus maze test	Yes	Yes
	Light/dark transition test	No	Yes
Social behavior deficit	Social interaction test in novel environment	No	Yes
	Three-chamber social approach test	No	Unknown
	Home-cage social interaction test	Yes	Unknown
Perseveration	Barnes maze test	Yes	Unknown
Repetitive behavior	Grooming test	No	Yes

## Supplementary Materials

Supplementary materials can be found at www.mdpi.com/1422-0067/18/9/1872/s1.

## Abbreviations

ASD	Autism spectrum disorder
hKO	Hetero knockout
