# Supplementary material for: Arid1b Haploinsufficiency Causes Abnormal Brain Gene Expression and Autism-Related Behaviors in Mice
Source: Int J Mol Sci. 2017 Aug 30;18(9):1872. doi: 10.3390/ijms18091872 (PMC5618521; doi:10.3390/ijms18091872)
Supplement: Supplementary file 1 [file ijms-18-01872-s001.pdf]

# Figure S1

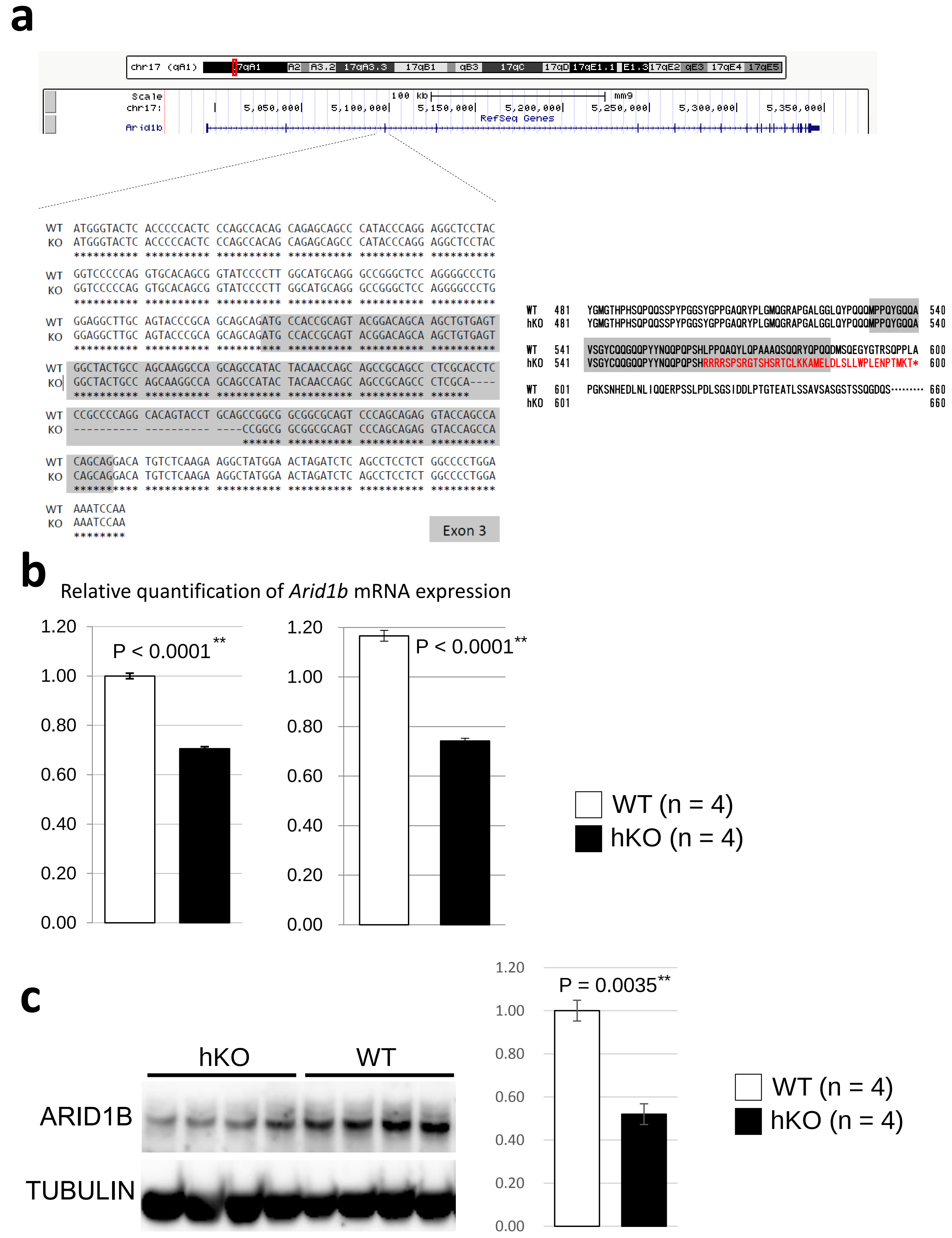

a
chr17 (qA1)
7qA1 A2 A3.2 17qA3.3 17qB1 qB3 17qC 17qD 17qE1.1 E1.3 17qE2 qE3 17qE4 17qE5
Scale
100 kb
mm9
chr17:
5,050,000| 5,100,000| 5,150,000| 5,200,000| 5,250,000| 5,300,000| 5,350,000|
RefSeq Genes
Arid1b
WT ATGGGTACTC ACCCCCACTC CCAGCCACAG CAGAGCAGCC CATACCCAGG AGGCTCCTAC
KO ATGGGTACTC ACCCCCACTC CCAGCCACAG CAGAGCAGCC CATACCCAGG AGGCTCCTAC
WT GGTCCCCCAG GTGCACAGCG GTATCCCCTT GGCATGCAGG GCCGGGCTCC AGGGGCCCTG
KO GGTCCCCCAG GTGCACAGCG GTATCCCCTT GGCATGCAGG GCCGGGCTCC AGGGGCCCTG
WT GGAGGCTTGC AGTACCCGCA GCAGCAGATG CCACCGCAGT ACGGACAGCA AGCTGTGAGT
KO GGAGGCTTGC AGTACCCGCA GCAGCAGATG CCACCGCAGT ACGGACAGCA AGCTGTGAGT
WT GGCTACTGCC AGCAAGGCCA GCAGCCATAC TACAACCAGC AGCCGCAGCC CTCGCACCTC
KO GGCTACTGCC AGCAAGGCCA GCAGCCATAC TACAACCAGC AGCCGCAGCC CTCGCA----
WT CCGCCCCAGG CACAGTACCT GCAGCCGGCG GCGGCGCAGT CCCAGCAGAG GTACCAGCCA
KO ---------- ---------- ----CCGGCG GCGGCGCAGT CCCAGCAGAG GTACCAGCCA
WT CAGCAGGACA TGTCTCAAGA AGGCTATGGA ACTAGATCTC AGCCTCCTCT GGCCCCTGGA
KO CAGCAGGACA TGTCTCAAGA AGGCTATGGA ACTAGATCTC AGCCTCCTCT GGCCCCTGGA
WT AAATCCAA
KO AAATCCAA
Exon 3
WT 481 YGMGTHPHSQPQQSSPYPGGSYGPPGAQRYPLGMQGRAPGALGGLQYPQQQMPPQYGQQA 540
hKO 481 YGMGTHPHSQPQQSSPYPGGSYGPPGAQRYPLGMQGRAPGALGGLQYPQQQMPPQYGQQA 540
WT 541 VSGYCQQGQQPYYNQQPQPSHLPPQAQYLQPAAAQSQQRYQPQQDMSQEGYGTRSQPPLA 600
hKO 541 VSGYCQQGQQPYYNQQPQPSHRRRRSPSRGTSHSRTCLKKAMELDLSLLWPLENPTMKT* 600
WT 601 PGKSNHEDLNLIQQERPSSLPDLSGSIDDLPTGTEATLSSAVSASGSTSSQGDQS········· 660
hKO 601 660
b
Relative quantification of *Arid1b* mRNA expression
1.20
1.00
0.80
0.60
0.40
0.20
0.00
P < 0.0001**
P < 0.0001**
WT (n = 4)
hKO (n = 4)
c
hKO
WT
ARID1B
TUBULIN
P = 0.0035**
WT (n = 4)
hKO (n = 4)

**Figure S1.** Generation of *Arid1b* hKO mice. (a) Deletion of the exon 3 region of the *Arid1b* gene results in premature termination. (b) Quantification of Arid1b mRNA in hKO mouse brains by RNA-Seq (left) and RT-PCR (right). (c) Quantification of ARID1B protein in hKO mouse brain by western assay. Data are expressed as the mean ± SEM (WT, $n = 4$; *Arid1b* hKO, $n = 4$). $p$-values were calculated using Student's two-tailed $t$-test.

# Figure S2

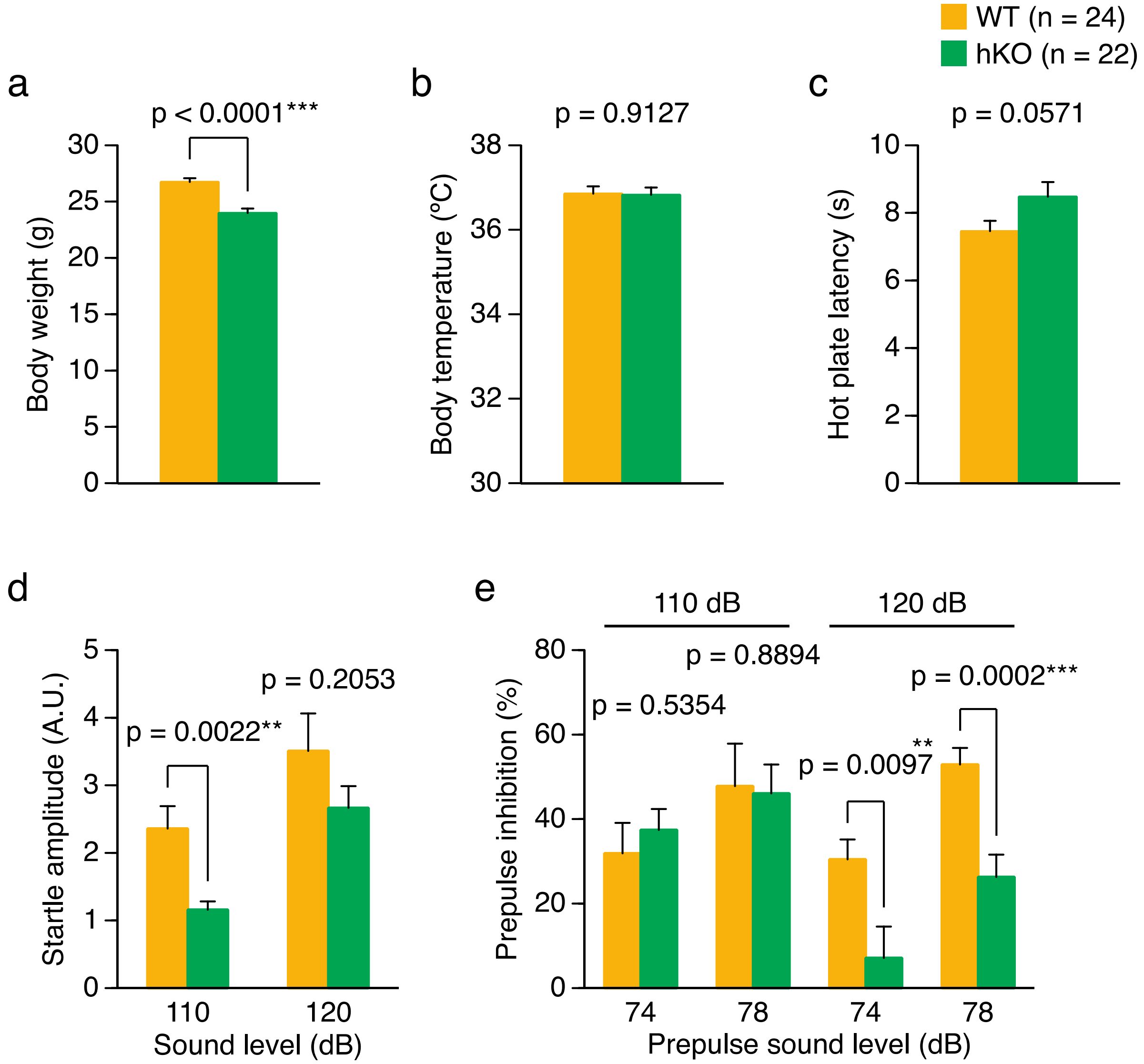


**Figure S2.** Physical characteristics and sensory functions in *Arid1b* hKO mice. (a) Body weight. (b) Body temperature. (c) Hot-plate latency. (d, e) Prepulse inhibition test. (d) Startle response amplitude. (e) Percentage of prepulse inhibition. Data are expressed as the mean ± SEM (WT, $n$ = 24; *Arid1b* hKO, $n$ = 22). Significant differences between genotypes are indicated by ** $p < 0.01$ and *** $p < 0.001$, as determined by Student's two-tailed $t$-test.

# Figure S3

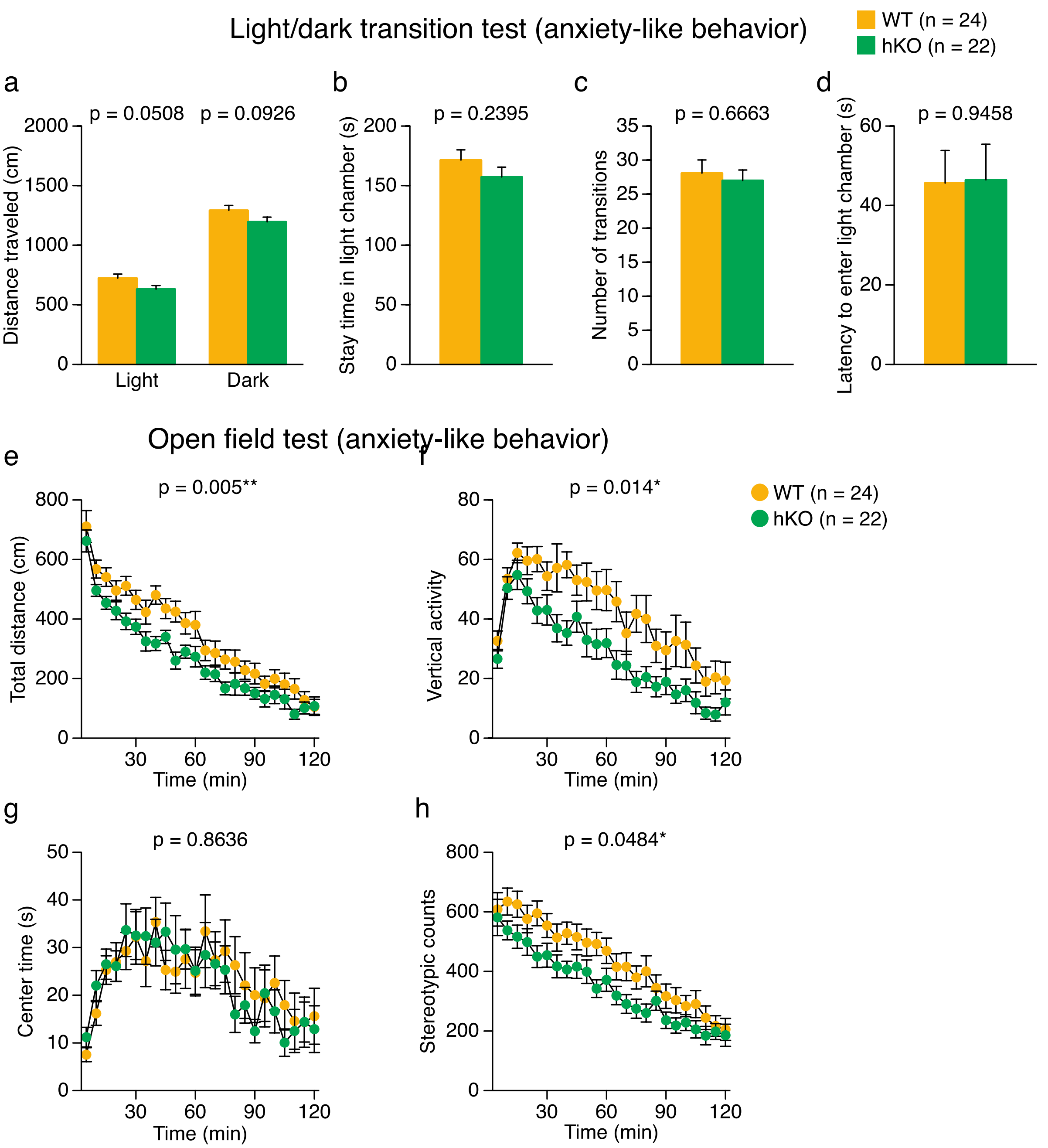


**Figure S3.** Locomotor activity and anxiety-like behavior in *Arid1b* hKO mice. (a–d) Light/dark transition test. (a) Distance traveled. (b) Stay time in the light chamber. (c) Number of transitions between light and dark chambers. (d) Latency of entering the light chamber. (e–h) Open field test. (e) Distance traveled. (f) Vertical activity. (g) Stay time in center area. (h) Stereotypic behavior counts. Data are expressed as the mean ± SEM (WT, $n$ = 24; *Arid1b* hKO, $n$ = 22). Significant differences between genotypes are indicated by * $p < 0.05$ and ** $p < 0.01$, as determined by Student's two-tailed $t$-test (a–d) or two-way repeated-measures ANOVA (e–h).

# Figure S4

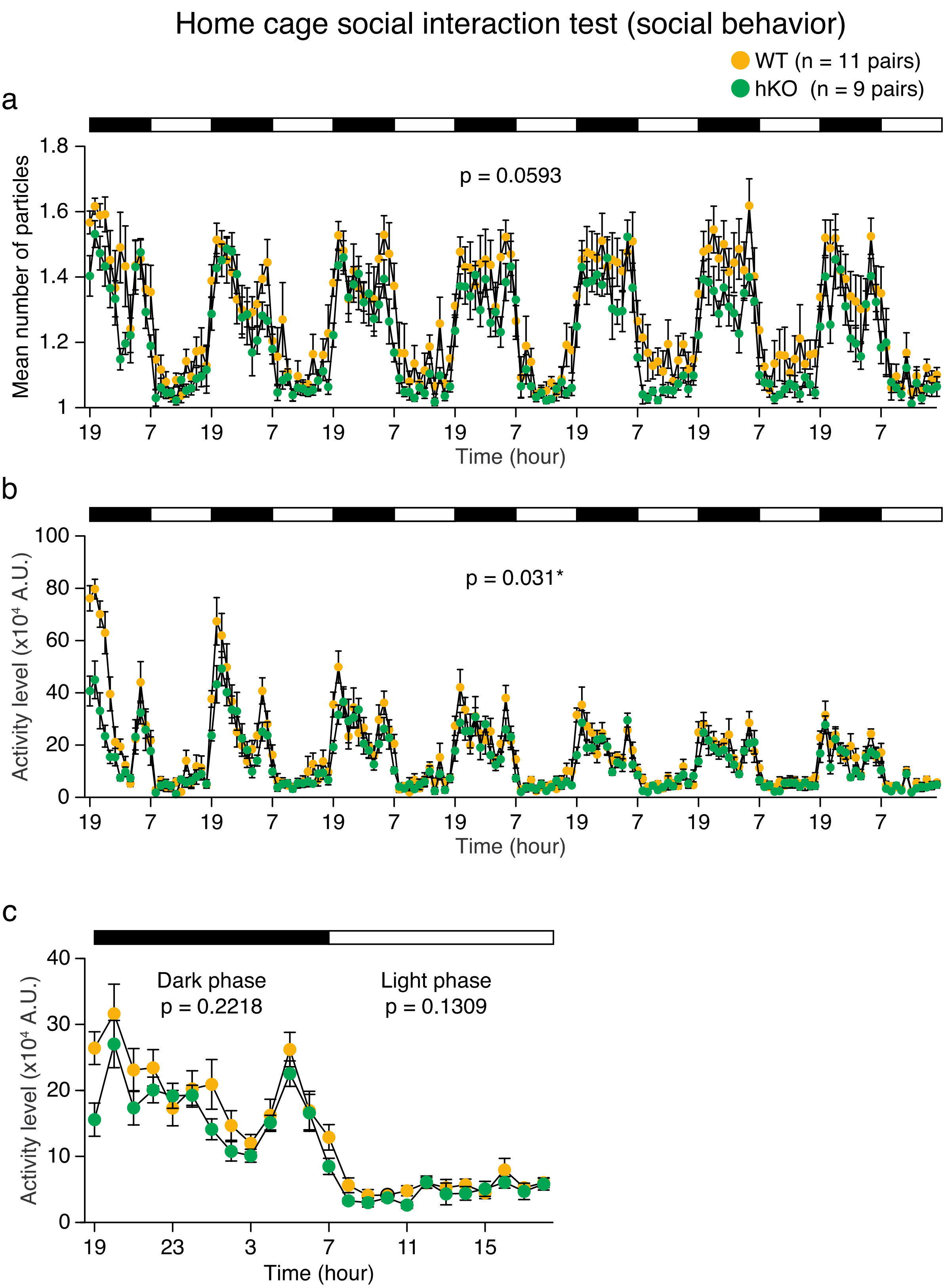


**Figure S4.** Locomotor activity and social behavior in a home cage in *Arid1b* hKO mice. (a–c) Home cage social interaction test. (a) Mean number of particles detected in the home cage during each hour of 1 week. Two particles indicate that the mice were not in contact with each other, and one particle indicates contact between the two mice. (b) Activity levels in pairs of mice. (c) Mean activity levels averaged over the last 3 days. Data are expressed as the mean ± SEM (WT, $n = 11$; *Arid1b* hKO, $n = 9$). Significant differences between genotypes are indicated by $*p < 0.05$ and $**p < 0.01$, determined by two-way repeated-measures ANOVA.

# Figure S5

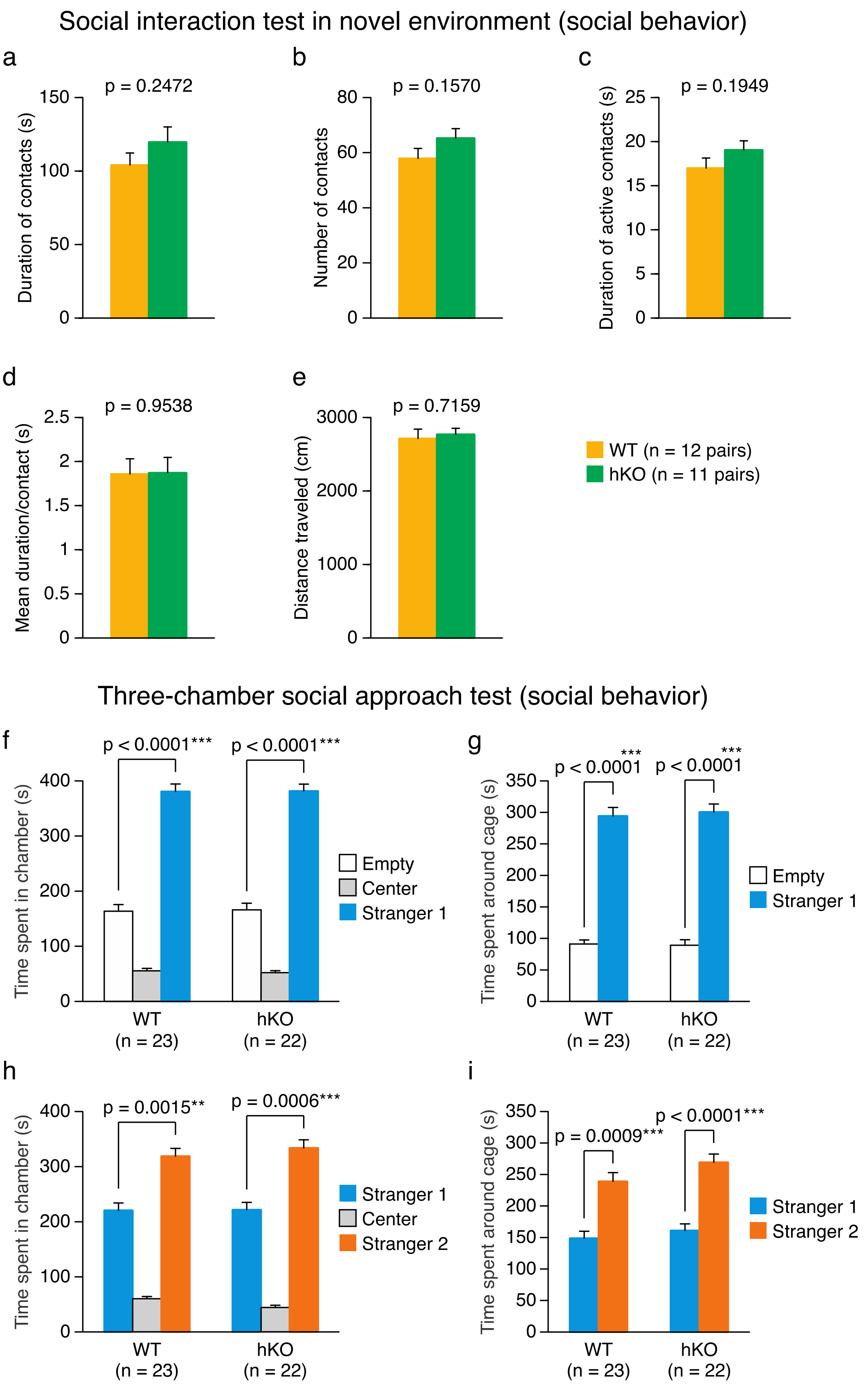

Social interaction test in novel environment (social behavior)
a
p = 0.2472
Duration of contacts (s)
150
100
50
0
b
p = 0.1570
Number of contacts
80
60
40
20
0
c
p = 0.1949
Duration of active contacts (s)
25
20
15
10
5
0
d
p = 0.9538
Mean duration/contact (s)
2.5
2
1.5
1
0.5
0
e
p = 0.7159
Distance traveled (cm)
3000
2000
1000
0
WT (n = 12 pairs)
hKO (n = 11 pairs)
Three-chamber social approach test (social behavior)
f
p < 0.0001***
p < 0.0001***
Time spent in chamber (s)
400
300
200
100
0
WT
(n = 23)
hKO
(n = 22)
Empty
Center
Stranger 1
g
***
p < 0.0001
***
p < 0.0001
Time spent around cage (s)
350
300
250
200
150
100
50
0
WT
(n = 23)
hKO
(n = 22)
Empty
Stranger 1
h
p = 0.0015**
p = 0.0006***
Time spent in chamber (s)
400
300
200
100
0
WT
(n = 23)
hKO
(n = 22)
Stranger 1
Center
Stranger 2
i
p = 0.0009***
p < 0.0001***
Time spent around cage (s)
350
300
250
200
150
100
50
0
WT
(n = 23)
hKO
(n = 22)
Stranger 1
Stranger 2

**Figure S5.** Social behavior of *Arid1b* hKO mice in the open field and in the three-chamber apparatus. (a–e) Social interaction test in a novel environment. (a) Duration of contacts. (b) Number of contacts. (c) Duration of active contacts. (d) Mean duration per contact. (e) Total distance traveled. (f–i) Three-chamber social approach test. (f) Time spent in chamber with an empty cage, center chamber, and chamber with a cage containing a stranger mouse (stranger 1). (g) Time spent around the empty cage and the cage with stranger 1. (h) Time spent in the chamber with the cage containing stranger 1, center chamber, and chamber with a cage containing a novel unfamiliar mouse (stranger 2). (g) Time spent around the cage containing stranger 1 and the cage containing stranger 2. Data from the social interaction test (WT, $n$ = 12 pairs; *Arid1b* hKO, $n$ = 11 pairs) and three-chamber social approach test (WT, $n$ = 23; *Arid1b* hKO, $n$ = 22) are expressed as the mean ± SEM. Significant differences between genotypes are indicated with ** $p < 0.01$ and *** $p < 0.001$, as determined by Student's two-tailed $t$-test (a–e) or unpaired $t$-test (f–i).

# Figure S6

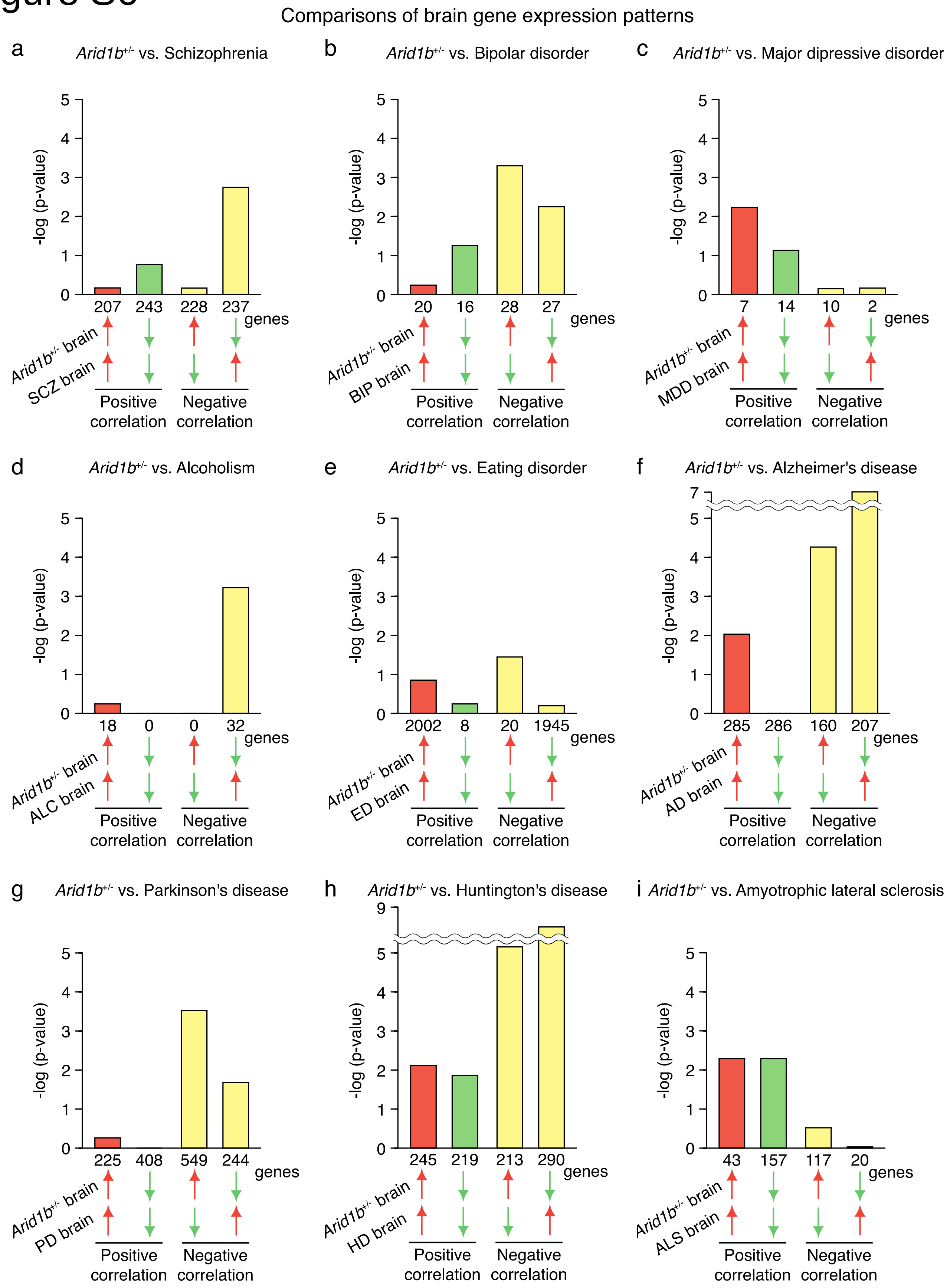


**Figure S6.** Comparison of brain gene expression patterns in *Arid1b* hKO mice with other genomic data. (a) Comparison of brain gene expression patterns in *Arid1b* hKO mice with those in schizophrenia, (b) bipolar disorder, (c) depressive disorder, (d) alcoholism, (e) eating disorder, (f) Alzheimer's disease, (g) Parkinson's disease, (h) Huntington's disease and (i) amyotrophic lateral sclerosis patients.

Figure S7

Malformed brain in 5% of *Arid1b* hKO mice

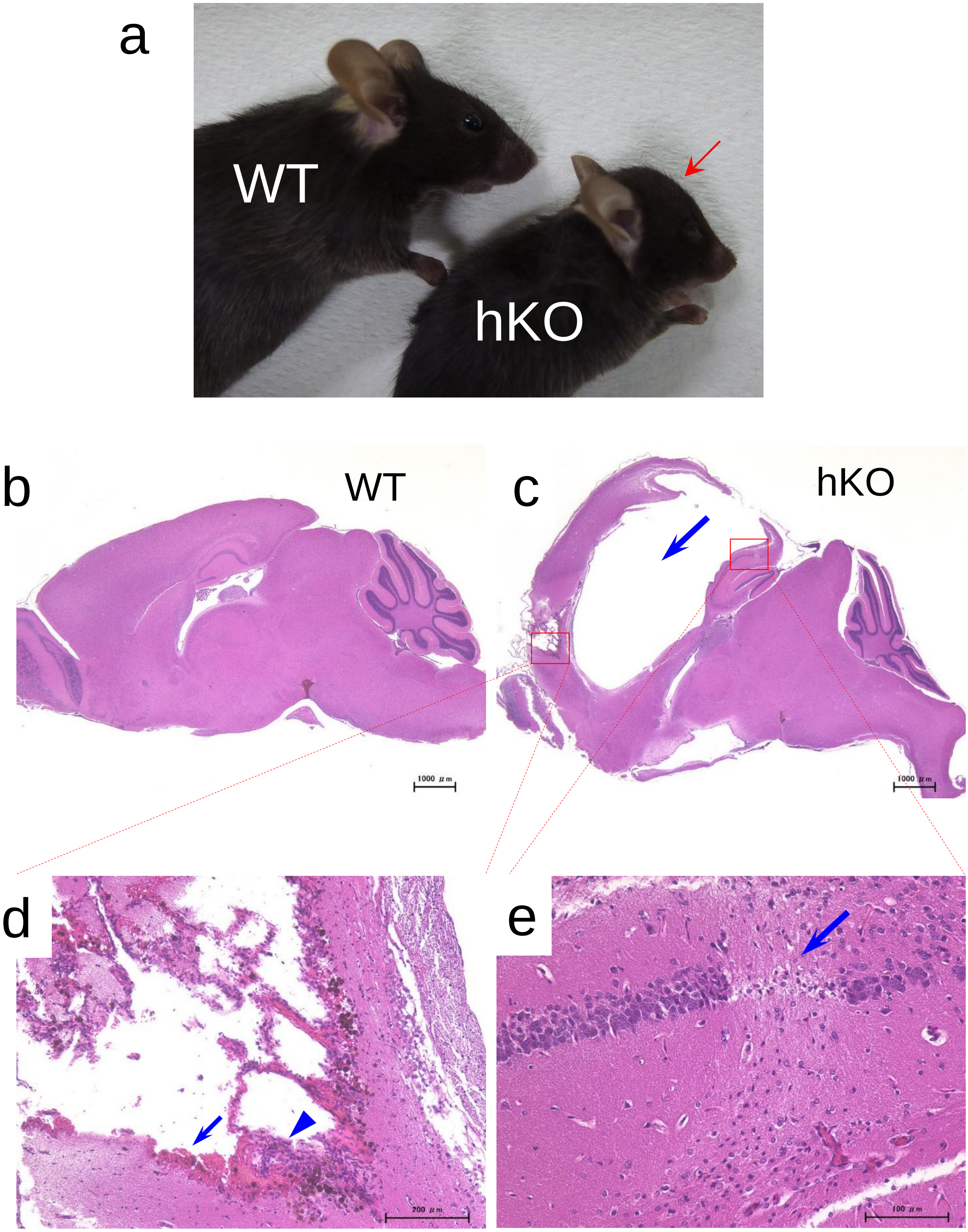

**Figure S7.** Appearance of *Arid1b* hKO mouse with malformed brain and wild type mouse with normal brain. (a) A mouse with an inflated head (right, red arrow) and a normal littermate (left) are *Arid1b* hKO and wild type, respectively. (b) Wild-type mouse brain (sagittal section) stained with hematoxylin–eosin. (c) Section of *Arid1b* hKO brain. Enlarged lateral ventricle (arrow) and some malformations (red frames) are indicated. (d) Enlargement of the left-hand framed portion of (c), showing an abnormal cavity (arrow) and new vascularization (arrow head). (e) Enlargement of the right-hand framed portion of (c), showing corruption in the pyramidal cell layer of the hippocampus (arrow).

**Table S1: A behavioral test battery in Arid1b hKO mice.**

| Order | Test | Age (weeks) | Figure |
|---|---|---|---|
| 1 | General health and neurological screen | 10-13 | Figure 2a, b and S2a-c |
| 2 | Light/dark transition test | 10-13 | Figure S3a-d |
| 3 | Open field test | 11-13 | Figure S3e-h |
| 4 | Elevated plus maze test | 11-14 | Figure 1a-d |
| 5 | Hot plate test | 12-14 | Figure S2c |
| 6 | Social interaction test | 12-14 | Figure S5a-e |
| 7 | Rotarod test | 12-15 | Figure 1c |
| 8 | Three-chamber social approach test | 13-15 | Figure S5f-i |
| 9 | Prepulse inhibition test | 13-16 | Figure S2d, e |
| 10 | Porsolt forced swim test | 13-16 | Figure 3g, h |
| 11 | Beam test | 16-19 | Figure 2d-h |
| 12 | Barnes maze test | 16-23 | Figure 1g, h |
| 13 | Fear conditioning test | 33-40 | Figure 3a-f |
| 14 | Home cage social interaction test | 39-42 | Figure 1e, f and S4a-c |

Supplementary Table S2. Microarray and RNA-seq datasets used in the brain gene expression pattern analysis.

| Dataset | Accession number | Brain sample | Figure |
|---|---|---|---|
| Autism spectrum disorder | GSE38322 | Occipital lobe of autistic patients | Figure 4a |
| Chd8 heterozygous knockout mice | DRA003116 | Whole brain of adult male Chd8 heterozygous knockout mice | Figure 4b |
| Fast-spiking interneurons in neonatal mouse brain | GSE17806 | Fast-spiking interneurons isolated from the somatosensory cortex of mice at 7 days old | Figure 4c |
| Schizophrenia (SCZ) | GSE53987 | Prefrontal cortex (BA46) tissues of female schizophrenia patients | Supplementary figure 6a |
| Bipolar disorder (BIP) | GSE35978 | Parietal cortex of patients with bipolar disorder | Supplementary figure 6b |
| Major depressive disorder (MDD) | GSE54575 | Orbital ventral prefrontal cortices of major depressive disorder patients | Supplementary figure 6c |
| Alcoholism (ALC) | GSE49376 | Prefrontal cortex of patients with alcoholism | Supplementary figure 6d |
| Eating disorder (ED) | GSE60190 | Prefrontal cortex of patients with eating disorder | Supplementary figure 6e |
| Obsessive-compulsive disorder | No data available | | |
| Panic disorder | No data available | | |
| Anxiety disorder | No data available | | |
| Posttraumatic stress disorder | No data available | | |
| Alzheimer's disease (AD) | GSE48350 | Post central gyruses of Alzheimer's disease patients | Supplementary figure 6f |
| Parkinson's disease (PD) | GSE8397 | Superior frontal gyrus of Parkinson's disease patients | Supplementary figure 6g |
| Huntington's disease (HD) | GSE79666 | Motor cortices of patients with Huntington's disease | Supplementary figure 6h |
| Amyotrophic lateral sclerosis (ALS) | GSE67196 | Frontal cortices of patients with amyotrophic lateral sclerosis | Supplementary figure 6i |
| Spinocerebellar disease | No data available | | |
| Multiple system atrophy | No data available | | |
| Progressive supranuclear ophthalmoplegia | No data available | | |

Supplementary Table S3. Statistical analyses of behavioral data.

| Test | Measure | Figure | t-test | ANOVA | | |
|---|---|---|---|---|---|---|
| | | | | Genotype | Trial/Time/Block/Session | Genotype × Trial/Time/Block/Session |
| Physical characteristics and neurological screen | Body weight (g) | Figure | $t_{44}$ = 5.44, p < 0.0001 | | | |
| | Body temperature (ºC) | Figure | $t_{44}$ = 0.11, p = 0.9127 | | | |
| | Grip strength (N) | Figure | $t_{44}$ = 2.21, p = 0.0325 | | | |
| | Wire hang latency (s) | Figure | $t_{44}$ = 3.16, p = 0.0029 | | | |
| Hot plate test | Hot plate latency (s) | Figure | $t_{44}$ = 1.95, p = 0.0571 | | | |
| Rotarod test | Rotarod latency (s) | Figure | | $F_{1,44}$ = 20.58, p < 0.0001 | $F_{5,220}$ = 20.39, p < 0.0001 | $F_{5,220}$ = 0.65, p = 0.6630 |
| Beam test | Moving speed (cm/s) on wide beam | Figure | | $F_{1,21}$ = 9.94, p = 0.0048 | $F_{5,105}$ = 23.76, p < 0.0001 | $F_{5,105}$ = 0.66, p = 0.6553 |
| | Moving speed (cm/s) on narrow beam | Figure | | $F_{1,21}$ = 11.22, p = 0.0030 | $F_{5,105}$ = 4.37, p = 0.0012 | $F_{5,105}$ = 1.32, p = 0.2604 |
| | Number of movements on wide beam | Figure | | $F_{1,21}$ = 0.27, p = 0.6077 | $F_{5,105}$ = 2.36, p = 0.0453 | $F_{5,105}$ = 0.34, p = 0.8877 |
| | Number of movements on narrow beam | Figure | | $F_{1,21}$ = 5.68, p = 0.0267 | $F_{5,105}$ = 1.84, p = 0.1106 | $F_{5,105}$ = 0.54, p = 0.7445 |
| | Total duration of movements (s) on wide beam | Figure | | $F_{1,21}$ = 13.11, p = 0.0016 | $F_{5,105}$ = 23.43, p < 0.0001 | $F_{5,105}$ = 0.28, p = 0.9215 |
| | Total duration of movements (s) on narrow beam | Figure | | $F_{1,21}$ = 14.77, p = 0.0009 | $F_{5,105}$ = 5.06, p = 0.0003 | $F_{5,105}$ = 1.37, p = 0.2433 |
| | Latency (s) on wide beam | Figure | | $F_{1,21}$ = 5.43, p = 0.0299 | $F_{5,105}$ = 3.45, p = 0.0063 | $F_{5,105}$ = 0.59, p = 0.7059 |
| | Latency (s) on narrow beam | Figure | | $F_{1,21}$ = 2.13, p = 0.1591 | $F_{5,105}$ = 2.21, p = 0.0587 | $F_{5,105}$ = 0.16, p = 0.9763 |
| | Number of slips on wide beam | Figure | | $F_{1,21}$ = 11.98, p = 0.0023 | $F_{5,105}$ = 4.39, p = 0.0011 | $F_{5,105}$ = 2.24, p = 0.0557 |
| | Number of slips on narrow beam | Figure | | $F_{1,21}$ = 20.65, p = 0.0002 | $F_{5,105}$ = 1.64, p = 0.1551 | $F_{5,105}$ = 1.14, p = 0.3433 |
| Open field test | Distance traveled (cm) | Figure | | $F_{1,44}$ = 8.74, p = 0.0050 | $F_{23,1012}$ = 70.66, p < 0.0001 | $F_{23,1012}$ = 1.05, p = 0.4036 |
| | Vertical activity | Figure | | $F_{1,44}$ = 6.54, p = 0.0140 | $F_{23,1012}$ = 29.84, p < 0.0001 | $F_{23,1012}$ = 1.20, p = 0.2358 |
| | Center time (s) | Figure | | $F_{1,44}$ = 0.03, p = 0.8636 | $F_{23,1012}$ = 5.98, p < 0.0001 | $F_{23,1012}$ = 0.70, p = 0.8530 |
| | Stereotypic counts | Figure | | $F_{1,44}$ = 4.12, p = 0.0484 | $F_{23,1012}$ = 49.56, p < 0.0001 | $F_{23,1012}$ = 1.04, p = 0.4144 |
| Light/dark transition test | Distance traveled (cm) in light chamber | Figure | $t_{44}$ = 2.01, p = 0.0508 | | | |
| | Distance traveled (cm) in dark chamber | Figure | $t_{44}$ = 1.72, p = 0.0926 | | | |
| | Latency to light chamber (s) | Figure | $t_{35}$ = 0.28, p = 0.7827 | | | |
| | Time spent in light chamber (s) | Figure | $t_{44}$ = 1.19, p = 0.2395 | | | |
| | Number of transitions | Figure | $t_{44}$ = 0.43, p = 0.6663 | | | |
| Elevated plus maze test | Distance traveled (cm) | Figure | $t_{43}$ = 2.09, p = 0.0429 | | | |
| | Number of entries | Figure | $t_{43}$ = 2.36, p = 0.0231 | | | |
| | Entries into open arms (%) | Figure | $t_{43}$ = 2.82, p = 0.0072 | | | |
| | Time on open arms (%) | Figure | $t_{43}$ = 2.31, p = 0.0259 | | | |
| Social interaction test | Distance traveled (cm) | Figure | $t_{21}$ = 0.37, p = 0.7159 | | | |
| | Number of contacts | Figure | $t_{21}$ = 1.47, p = 0.1570 | | | |
| | Total duration of contacts (s) | Figure | $t_{21}$ = 1.19, p = 0.2472 | | | |
| | Total duration of active contacts (s) | Figure | $t_{21}$ = 1.34, p = 0.1949 | | | |
| | Mean duration of contact (s) | Figure | $t_{21}$ = 0.06, p = 0.9538 | | | |
| Three-chamber social approach test (Sociability test) | Time spent in chamber (s) in WT | Figure | $t_{22}$ = 8.62, p < 0.0001 | | | |
| | Time spent in chamber (s) in hKO | Figure | $t_{21}$ = 8.93, p < 0.0001 | | | |
| | Time spent around cage (s) in WT | Figure | $t_{22}$ = 11.63, p < 0.0001 | | | |
| | Time spent around cage (s) in hKO | Figure | $t_{21}$ = 10.77, p < 0.0001 | | | |
| Three-chamber social approach test (Social novelty preference test) | Time spent in chamber (s) in WT | Figure | $t_{22}$ = 3.62, p = 0.0015 | | | |
| | Time spent in chamber (s) in hKO | Figure | $t_{21}$ = 4.05, p = 0.0006 | | | |
| | Time spent around cage (s) in WT | Figure | $t_{22}$ = 3.82, p = 0.0009 | | | |
| | Time spent around cage (s) in hKO | Figure | $t_{21}$ = 4.84, p < 0.0001 | | | |
| Home cage social interaction test | Activity level (A.U.) | Figure | | $F_{1,18}$ = 5.48, p = 0.0310 | $F_{167,3006}$ = 28.95, p < 0.0001 | $F_{167,3006}$ = 2.52, p < 0.0001 |
| | Average activity level (A.U.) during the last 3 days (light phase) | Figure | | $F_{1,18}$ = 2.50, p = 0.1309 | $F_{11,198}$ = 8.33, p < 0.0001 | $F_{11,198}$ = 1.00, p = 0.4433 |
| | Average activity level (A.U.) during the last 3 days (dark phase) | Figure | | $F_{1,18}$ = 1.60, p = 0.2218 | $F_{11,198}$ = 13.47, p < 0.0001 | $F_{11,198}$ = 1.56, p = 0.1146 |
| | Number of particles | Figure | | $F_{1,18}$ = 4.05, p = 0.0593 | $F_{167,3006}$ = 17.71, p < 0.0001 | $F_{167,3006}$ = 0.89, p = 0.8278 |
| | Average number of particles during the last 3 days (light phase) | Figure | | $F_{1,18}$ = 6.17, p = 0.0231 | $F_{11,198}$ = 4.08, p < 0.0001 | $F_{11,198}$ = 0.44, p = 0.9367 |
| | Average number of particles during the last 3 days (dark phase) | Figure | | $F_{1,18}$ = 3.11, p = 0.0949 | $F_{11,198}$ = 6.20, p < 0.0001 | $F_{11,198}$ = 0.61, p = 0.8188 |
| Startle response test | Startle response (110 dB) | Figure | $t_{44}$ = 3.26, p = 0.0022 | | | |
| | Startle response (120 dB) | Figure | $t_{44}$ = 1.29, p = 0.2053 | | | |
| Prepulse inhibition test | PPI (74-110 dB) | Figure | $t_{44}$ = 0.62, p = 0.5354 | | | |
| | PPI (78-110 dB) | Figure | $t_{44}$ = 0.14, p = 0.8894 | | | |
| | PPI (74-120 dB) | Figure | $t_{44}$ = 2.70, p = 0.0097 | | | |
| | PPI (78-120 dB) | Figure | $t_{44}$ = 4.09, p = 0.0002 | | | |
| Porsolt forced swim test | Immobility (%) on Day 1 | Figure | | $F_{1,42}$ = 3.90, p = 0.0550 | $F_{9,378}$ = 132.35, p < 0.0001 | $F_{9,378}$ = 2.29, p = 0.0164 |
| | Immobility (%) on Day 2 | Figure | | $F_{1,42}$ = 4.13, p = 0.0484 | $F_{9,378}$ = 21.21, p < 0.0001 | $F_{9,378}$ = 2.22, p = 0.0200 |
| | Distance traveled (cm) on Day 1 | Figure | | $F_{1,42}$ = 4.13, p = 0.0485 | $F_{9,378}$ = 188.60, p < 0.0001 | $F_{9,378}$ = 2.67, p = 0.0051 |
| | Distance traveled (cm) on Day 2 | Figure | | $F_{1,42}$ = 0.93, p = 0.3404 | $F_{9,378}$ = 23.29, p < 0.0001 | $F_{9,378}$ = 1.77, p = 0.0716 |
| Barnes maze test (acquisition) | Latency to target (s) | Figure | | $F_{1,40}$ = 2.87, p = 0.0980 | $F_{8,320}$ = 20.89, p < 0.0001 | $F_{8,320}$ = 0.23, p = 0.9842 |
| Barnes maze test (reversal) | Latency to target (s) | Figure | | $F_{1,40}$ = 4.78, p = 0.0347 | $F_{5,200}$ = 49.51, p < 0.0001 | $F_{5,200}$ = 2.16, p = 0.0595 |
| Barnes maze test (probe trial) | Time spent around target hole (s) | Figure | $t_{40}$ = 2.28, p = 0.0279 | | | |
| Fear conditioning test (Conditioning) | Freezing (%) | Figure | | $F_{1,40}$ = 9.23, p = 0.0042 | $F_{7,280}$ = 17.65, p < 0.0001 | $F_{7,280}$ = 6.01, p < 0.0001 |
| Fear conditioning test (Context test 1 day after conditioning) | Freezing (%) | Figure | | $F_{1,40}$ = 0.57, p = 0.4565 | $F_{4,160}$ = 5.04, p = 0.0007 | $F_{4,160}$ = 0.55, p = 0.6999 |
| Fear conditioning test (Cued test 1 day after conditioning, Pre-CS) | Freezing (%) | Figure | | $F_{1,40}$ = 6.76, p = 0.0130 | $F_{2,80}$ = 29.40, p < 0.0001 | $F_{2,80}$ = 1.59, p = 0.2101 |
| Fear conditioning test (Cued test 1 day after conditioning, CS) | Freezing (%) | Figure | | $F_{1,40}$ = 2.71, p = 0.1076 | $F_{2,80}$ = 21.31, p < 0.0001 | $F_{2,80}$ = 0.22, p = 0.8031 |
| Fear conditioning test (Context test 30 days after conditioning) | Freezing (%) | Figure | | $F_{1,40}$ = 0.03, p = 0.8576 | $F_{4,160}$ = 4.15, p = 0.0032 | $F_{4,160}$ = 0.75, p = 0.5568 |
| Fear conditioning test (Cued test 30 days after conditioning, Pre-CS) | Freezing (%) | Figure | | $F_{1,40}$ = 2.77, p = 0.1036 | $F_{2,80}$ = 9.46, p = 0.0002 | $F_{2,80}$ = 1.66, p = 0.1972 |
| Fear conditioning test (Cued test 30 days after conditioning, CS) | Freezing (%) | Figure | | $F_{1,40}$ = 5.97, p = 0.0191 | $F_{2,80}$ = 12.92, p < 0.0001 | $F_{2,80}$ = 0.23, p = 0.7970 |
| Fear conditioning test (footshock 1) | Distance traveled (cm) | Figure | | $F_{1,40}$ = 0.62, p = 0.4373 | $F_{14,560}$ = 40.72, p < 0.0001 | $F_{14,560}$ = 1.48, p = 0.1146 |
| Fear conditioning test (footshock 2) | Distance traveled (cm) | Figure | | $F_{1,40}$ = 1.95, p = 0.1705 | $F_{14,560}$ = 44.01, p < 0.0001 | $F_{14,560}$ = 1.63, p = 0.0658 |
| Fear conditioning test (footshock 3) | Distance traveled (cm) | Figure | | $F_{1,40}$ = 6.08, p = 0.0181 | $F_{14,560}$ = 46.07, p < 0.0001 | $F_{14,560}$ = 1.22, p = 0.2585 |

Table S4. Down regulrated genes in *Arid1b* hKO mice brain (fold change < -2, p < 0.05)

| fold change | Gene Symbol | Description |
|---|---|---|
| -8.785834 | Hoxb2 | homeobox B2 |
| -7.0651803 | Prl | prolactin |
| -5.208952 | Tg | thyroglobulin |
| -4.509183 | 9330111N05Rik | RIKEN cDNA 9330111N05 gene |
| -4.284863 | Podnl1 | podocan-like 1 |
| -4.2418866 | Pth2 | parathyroid hormone 2 |
| -4.225615 | Igf2bp1 | insulin-like growth factor 2 mRNA binding protein 1 |
| -4.1967397 | Gm2990 | predicted gene 2990 |
| -4.1946692 | 1700023A16Rik | RIKEN cDNA 1700023A16 gene |
| -4.0916605 | Ccl3 | chemokine (C-C motif) ligand 3 |
| -3.8462672 | LOC100503966 | uncharacterized LOC100503966 |
| -3.5904546 | Cryaa | crystallin, alpha A |
| -3.5482562 | Gm20199 | predicted gene, 20199 |
| -3.510942 | Lin28a | lin-28 homolog A (C. elegans) |
| -3.320444 | Abcb11 | ATP-binding cassette, sub-family B (MDR/TAP), member 11 |
| -3.3167024 | C1qtnf3 | C1q and tumor necrosis factor related protein 3 |
| -3.2939663 | Hoxd3 | homeobox D3 |
| -3.2942612 | 4932411G14Rik | RIKEN cDNA 4932411G14 gene |
| -3.276335 | Pax8 | paired box gene 8 |
| -3.2846475 | Gm5665 | predicted gene 5665 |
| -3.2428174 | Hbq1b | hemoglobin, theta 1B |
| -3.2238915 | Gm20337 | predicted gene, 20337 |
| -3.135054 | 4933405L10Rik | RIKEN cDNA 4933405L10 gene |
| -3.0332363 | B230208H11Rik | RIKEN cDNA B230208H11 gene |
| -3.0280447 | Gm19673 | predicted gene, 19673 |
| -2.99006 | Agrp | agouti related protein |
| -2.9797118 | Gm15941 | predicted gene 15941 |
| -2.9233184 | 4833417C18Rik | RIKEN cDNA 4833417C18 gene |
| -2.8478506 | Acmsd | amino carboxymuconate semialdehyde decarboxylase |
| -2.8263907 | Guca1a | guanylate cyclase activator 1a (retina) |
| -2.8012562 | Ptk6 | PTK6 protein tyrosine kinase 6 |
| -2.7921495 | Ppyr1 | pancreatic polypeptide receptor 1 |
| -2.7751737 | Cdca3 | cell division cycle associated 3 |
| -2.7706063 | Vgll2 | vestigial like 2 homolog (Drosophila) |
| -2.7611718 | Lipm | lipase, family member M |
| -2.7061338 | Zfp345 | zinc finger protein 345 |
| -2.6982725 | Mboat4 | membrane bound O-acyltransferase domain containing 4 |
| -2.6775281 | Naa11 | N(alpha)-acetyltransferase 11, NatA catalytic subunit |
| -2.6551392 | Neurog1 | neurogenin 1 |
| -2.5700898 | Tc2n | tandem C2 domains, nuclear |
| -2.5540054 | Has2as | HAS2 antisense RNA (non-protein coding) |
| -2.4242957 | Gm9725 | predicted gene 9725 |
| -2.406591 | Cyp2d26 | cytochrome P450, family 2, subfamily d, polypeptide 26 |
| -2.3968318 | Snora75 | small nucleolar RNA, H/ACA box 75 |
| -2.3988662 | Irx6 | Iroquois related homeobox 6 (Drosophila) |
| -2.3923612 | Synb | syncytin b |
| -2.3772993 | E130218I03Rik | RIKEN cDNA E130218I03 gene |
| -2.364054 | Clic3 | chloride intracellular channel 3 |
| -2.337002 | Hgfac | hepatocyte growth factor activator |
| -2.3373454 | Npm2 | nucleophosmin/nucleoplasmin 2 |
| -2.3313246 | Mir23b | microRNA 23b |
| -2.2768116 | Gm13154 | predicted gene 13154 |
| -2.2683592 | Slc5a4a | solute carrier family 5, member 4a |
| -2.2600186 | Tusc5 | tumor suppressor candidate 5 |
| -2.203397 | Wfdc3 | WAP four-disulfide core domain 3 |
| -2.1879494 | Napsa | napsin A aspartic peptidase |
| -2.1738834 | Gm19557 | predicted gene, 19557 |
| -2.1692603 | Il27 | interleukin 27 |
| -2.1598554 | Rhoh | ras homolog gene family, member H |
| -2.1567962 | Epb4.2 | erythrocyte protein band 4.2 |
| -2.1499667 | Best3 | bestrophin 3 |
| -2.1152246 | Sbk2 | SH3-binding domain kinase family, member 2 |
| -2.120248 | Ccdc63 | coiled-coil domain containing 63 |
| -2.1180272 | Ccdc152 | coiled-coil domain containing 152 |
| -2.1040165 | Tepp | testis, prostate and placenta expressed |
| -2.0983396 | Muc1 | mucin 1, transmembrane |
| -2.0994968 | Fabp4 | fatty acid binding protein 4, adipocyte |
| -2.0862854 | Fam159b | family with sequence similarity 159, member B |
| -2.0938795 | Ly9 | lymphocyte antigen 9 |
| -2.0866616 | Igsf6 | immunoglobulin superfamily, member 6 |

Table S5. Up regulrated genes in *Arid1b* hKO mice brain (fold change < −2, p < 0.05)

| fold change | Gene Symbol | Description |
|---|---|---|
| 6.911925 | Areg | amphiregulin |
| 5.0602455 | Gbp8 | guanylate-binding protein 8 |
| 4.7839174 | Klri2 | killer cell lectin-like receptor family I member 2 |
| 4.435064 | Zp2 | zona pellucida glycoprotein 2 |
| 4.392006 | A730018C14Rik | RIKEN cDNA A730018C14 gene |
| 4.384695 | F630028O10Rik | RIKEN cDNA F630028O10 gene |
| 4.1315637 | Gdf7 | growth differentiation factor 7 |
| 4.08308 | Gm12408 | predicted gene 12408 |
| 4.00279 | Bpifa1 | BPI fold containing family A, member 1 |
| 3.9308658 | Klf14 | Kruppel-like factor 14 |
| 3.7878077 | Fgfr4 | fibroblast growth factor receptor 4 |
| 3.7767737 | Gm2518 | interferon induced transmembrane protein 2 pseudogene |
| 3.6329799 | Otop3 | otopetrin 3 |
| 3.5988166 | 1700123I01Rik | RIKEN cDNA 1700123I01 gene |
| 3.5129895 | Mesp2 | mesoderm posterior 2 |
| 3.5110056 | Emilin3 | elastin microfibril interfacer 3 |
| 3.5066862 | Gm14492 | predicted gene 14492 |
| 3.4818408 | Tmem45b | transmembrane protein 45b |
| 3.40611 | Mogat1 | monoacylglycerol O-acyltransferase 1 |
| 3.383388 | Fbxo43 | F-box protein 43 |
| 3.3563833 | Gpr84 | G protein-coupled receptor 84 |
| 3.3490303 | Krt83 | keratin 83 |
| 3.3343937 | Myo15b | myosin XVB |
| 3.3041756 | Usp50 | ubiquitin specific peptidase 50 |
| 3.2955153 | Gm11961 | predicted gene 11961 |
| 3.289348 | Pax5 | paired box gene 5 |
| 3.2450645 | Slc6a14 | solute carrier family 6 (neurotransmitter transporter), member 14 |
| 3.222701 | 2010001M09Rik | RIKEN cDNA 2010001M09 gene |
| 3.2140906 | Ffar3 | free fatty acid receptor 3 |
| 3.1673195 | Mmp8 | matrix metallopeptidase 8 |
| 3.1610575 | Cyp24a1 | cytochrome P450, family 24, subfamily a, polypeptide 1 |
| 3.147995 | Aqp2 | aquaporin 2 |
| 3.0993717 | 1700120B22Rik | RIKEN cDNA 1700120B22 gene |
| 3.084226 | Slc25a31 | solute carrier family 25 (mitochondrial carrier; adenine nucleotide translocator), member 31 |
| 3.0516515 | Fbp1 | fructose bisphosphatase 1 |
| 3.0512648 | Cyp2c44 | cytochrome P450, family 2, subfamily c, polypeptide 44 |
| 2.9070935 | 4930519F16Rik | RIKEN cDNA 4930519F16 gene |
| 2.9061913 | Slc16a5 | solute carrier family 16 (monocarboxylic acid transporters), member 5 |
| 2.8569329 | A830012C17Rik | RIKEN cDNA A830012C17 gene |
| 2.835883 | Ces1f | carboxylesterase 1F |
| 2.7937973 | Olfr464 | olfactory receptor 464 |
| 2.7877383 | Cd244 | CD244 natural killer cell receptor 2B4 |
| 2.787456 | Hp | haptoglobin |
| 2.7588398 | BC024582 | cDNA sequence BC024582 |
| 2.745353 | 1600010M07Rik | RIKEN cDNA 1600010M07 gene |
| 2.7137992 | Ifi27l2b | interferon, alpha-inducible protein 27 like 2B |
| 2.7046108 | Gm12676 | predicted gene 12676 |
| 2.6633875 | Serpinb10-ps | serine (or cysteine) peptidase inhibitor, clade B (ovalbumin), member 10, pseudogene |
| 2.6597853 | Clec2i | C-type lectin domain family 2, member i |
| 2.6458151 | Tssk6 | testis-specific serine kinase 6 |
| 2.6296997 | Gm13403 | predicted gene 13403 |
| 2.6193464 | Gadl1 | glutamate decarboxylase-like 1 |
| 2.5904422 | Tmprss2 | transmembrane protease, serine 2 |
| 2.5618844 | Col9a1 | collagen, type IX, alpha 1 |
| 2.5561414 | Cilp | cartilage intermediate layer protein, nucleotide pyrophosphohydrolase |
| 2.511111 | Insm2 | insulinoma-associated 2 |
| 2.5058055 | Ceacam15 | carcinoembryonic antigen-related cell adhesion molecule 15 |
| 2.4807065 | Abp1 | amiloride binding protein 1 (amine oxidase, copper-containing) |
| 2.4807065 | 2310002J15Rik | RIKEN cDNA 2310002J15 gene |
| 2.473039 | Cldn22 | claudin 22 |
| 2.4644089 | Gm10334 | predicted gene 10334 |
| 2.4634347 | Plac8 | placenta-specific 8 |
| 2.4587035 | P2rx3 | purinergic receptor P2X, ligand-gated ion channel, 3 |
| 2.4282923 | Xirp1 | xin actin-binding repeat containing 1 |
| 2.415078 | Lif | leukemia inhibitory factor |
| 2.3905227 | Ttc22 | tetratricopeptide repeat domain 22 |
| 2.256361 | Ccr10 | chemokine (C-C motif) receptor 10 |
| 2.2426867 | Gm15104 | predicted gene 15104 |
| 2.2286832 | C1qtnf7 | C1q and tumor necrosis factor related protein 7 |
| 2.2283084 | Adamts7 | a disintegrin-like and metallopeptidase (reprolysin type) with thrombospondin type 1 motif, 7 |
| 2.2056947 | 1810064F22Rik | RIKEN cDNA 1810064F22 gene |
| 2.181652 | Prss55 | protease, serine, 55 |
| 2.1674907 | Klrb1f | killer cell lectin-like receptor subfamily B member 1F |
| 2.1520178 | Gyltl1b | glycosyltransferase-like 1B |
| 2.1451557 | Gm16497 | predicted gene 16497 |
| 2.1422753 | Atp6v1e2 | ATPase, H+ transporting, lysosomal V1 subunit E2 |
| 2.1388183 | LOC100503361 | transmembrane protein 95-like |
| 2.11306 | Timp1 | tissue inhibitor of metalloproteinase 1 |
| 2.090713 | Fam154a | family with sequence similarity 154, member A |
| 2.0536246 | Gm17281 | predicted gene, 17281 |
| 2.0510998 | Lpcat2b | lysophosphatidylcholine acyltransferase 2B |
| 2.048164 | Idi2 | isopentenyl-diphosphate delta isomerase 2 |
| 2.046321 | ORF63 | open reading frame 63 |
| 2.044373 | Pabpc4l | poly(A) binding protein, cytoplasmic 4-like |
| 2.0441408 | Fgf23 | fibroblast growth factor 23 |
| 2.0098748 | Gm9836 | predicted pseudogene 9836 |
